# LC-MS/MS based metabolomics and proteomics reveal candidate biomarkers and molecular mechanism of early IgA nephropathy

**DOI:** 10.1186/s12014-022-09387-5

**Published:** 2022-12-27

**Authors:** Di Zhang, Yaohan Li, Mingzhu Liang, Yan Liang, Jingkui Tian, Qiang He, Bingxian Yang, Juan Jin, Wei Zhu

**Affiliations:** 1 Nephrology Center, Department of Nephrology, Zhejiang Provincial People’s Hospital, Affiliated People’s Hospital, Hangzhou Medical College, Hangzhou, 310014 Zhejiang China; 2 College of Biomedical Engineering and Instrument Science, Zhejiang University, Hangzhou, 310027 Zhejiang China; 3 The Cancer Hospital of the University of Chinese Academy of Sciences (Zhejiang Cancer Hospital), Institute of Basic Medicine and Cancer (IBMC), Chinese Academy of Sciences, Hangzhou, 310002 Zhejiang China; 4 Department of Nephrology, The First Affiliated Hospital of Zhejiang Chinese Medical University (Zhejiang Provincial Hospital of Traditional Chinese Medicine), Hangzhou, 310000 Zhejiang China; 5 College of Life Science and Medicine, Zhejiang Sci-Tech University, Hangzhou, 310018 China

**Keywords:** Biomarker, IgA nephropathy, LC-MS/MS, Plasma proteomics, Plasma metabolomics

## Abstract

**Background:**

Immunoglobulin A nephropathy (IgAN), a globally common primary chronic glomerulopathy, is one of the leading causes of end-stage renal disease. However, the underlying mechanisms of IgAN have yet to be demonstrated. There were no adequate and reliable plasma biomarkers for clinical diagnosis, especially at the early stage. In the present study, integrative proteomics and metabolomics were aimed at exploring the mechanism of IgAN and identifying potential biomarkers.

**Methods:**

Plasma from IgAN and healthy individuals were collected and analyzed in a randomized controlled manner. Data-independent acquisition quantification proteomics and mass spectrometry based untargeted metabolomics techniques were used to profile the differentially expressed proteins (DEPs) and differentially abundant metabolites (DAMs) between two groups and identify potential biomarkers for IgAN from health at the early stage. Disease-related pathways were screened out by clustering and function enrichment analyses of DEPs and DAMs. And the potential biomarkers for IgAN were identified through the machine learning approach. Additionally, an independent cohort was used to validate the priority candidates by enzyme-linked immunosorbent assay (ELISA).

**Results:**

Proteomic and metabolomic analyses of IgAN plasma showed that the complement and the immune system were activated, while the energy and amino acid metabolism were disordered in the IgAN patients. PRKAR2A, IL6ST, SOS1, and palmitoleic acid have been identified as potential biomarkers. Based on the AUC value for the training and test sets, the classification performance was 0.994 and 0.977, respectively. The AUC of the external validation of the four biomarkers was 0.91.

**Conclusion:**

In this study, we combined proteomics and metabolomics techniques to analyze the plasma of IgAN patients and healthy individuals, constructing a biomarker panel, which could provide new insights and provide potential novel molecular diagnoses for IgAN.

**Supplementary Information:**

The online version contains supplementary material available at 10.1186/s12014-022-09387-5.

## Introduction

IgAN, an autoimmune disease, is the most common primary glomerulonephritis (GN) worldwide and a significant cause of chronic kidney disease (CKD) and kidney failure [1]. It has been reported that the incidence of IgAN varies from 0.2 to 5 per 100,000 individuals per year on different continents, which is the dominant cause of kidney failure in East Asian countries, representing up to half of the cases of primary GN in China [2]. Glomerular mesangial cell proliferation and extracellular matrix expansion in IgAN patients begin at an early stage of disease and progress to glomerular and interstitial sclerosis, with approximately 30% to 40% of patients progressing to end-stage renal disease (ESRD) within 20 years [3]. For patients who progressed to ESRD, the annual direct medical expenses for peritoneal dialysis, hemodialysis, and renal transplant were $11,019, $15,803, and $28,272, respectively, in China [4]. Meanwhile, IgAN patients suffered from impaired quality of life due to symptoms such as fatigue and depression. Although the original description of IgAN had been put forward for more than 50 years [5], the specific pathogenesis of IgAN was not entirely determined. It was because IgAN could follow highly diverse courses ranging from asymptomatic urinary abnormalities with the potential for spontaneous resolution to rapidly progressive GN (RPGN) with kidney failure. And there were few animal models for IgAN, particularly for its early stage, which severely hampers preclinical trials of new therapeutic agents [6]. Early symptoms of IgAN were mild and had usually been overlooked or underdiagnosed. A global perspective of IgAN suggested that early therapy may reduce the global burden of end-stage kidney disease caused by IgAN [7]. Patients with IgAN may benefit from early identification and aggressive intervention.

Currently, the diagnosis and risk stratification of IgAN was based on kidney biopsy [8]. A combination of pathologic grading and clinical characteristics had been used to develop a model that can predict the prognosis of IgAN [9]. However, renal puncture biopsies are invasive, not reproducible, and may result in severe complications such as bleeding [10], which cannot be an effective strategy to monitor the IgAN progression or early diagnosis [11]. Therefore, a non-invasive diagnosis approach was needed for early diagnosis of IgAN even before the onset of reduced eGFR [12]. Some biomarkers for IgAN diagnosis have been reported in recent years. Abnormal glycosylated (galactose deficient) immunoglobulin A1 (GD-IGA1) induced an autoimmune response and deposition of immune complexes in the kidney as possible pathogenesis for IgAN, which could be a potential, noninvasive biomarkers of IgAN [13, 14]. A prospective cohort study showed that the levels of platelet-derived growth factor DD (PDGF-DD) were explicitly increased in the serum of IgAN patients, particularly for those in the early stage of IgAN, so that it could serve as a biomarker of disease and/or disease activity [15]. However, Kidney Disease: Improving Global Outcomes (KDIGO) guidelines categorically stated in a practice point that, to date, there were still no validated blood or urine biomarkers for the diagnosis of IgAN [6]. Therefore, it is necessary to identify novel biomarkers that could be used for detecting early-stage IgAN. The present study was aimed at exploring the biological characteristics of IgAN and identifying the candidate biomarkers, which could contribute to a more holistic understanding of diseases and biological processes.

In recent decades, mass spectrometry (MS) based metabolomics and proteomics technologies have provided new perspectives on nephropathy studies. The applications of omics, including genomics, transcriptomics, proteomics, and metabolomics, can help us understand basic physiological processes and identify biomarkers [16, 17]. Recently, MS analysis of the urine proteome identified 14 different urinary protein fragments that belonged to the region of the connecting peptide of the total fetuin-A protein, demonstrating the association of fetuin-A peptides with impaired kidney function in T2DM patients, and can be used as markers for kidney disease detection [18]. Using a peptidome database, David G. et al. defined 273 biomarkers and built an SVM classification model to distinguish healthy subjects from individuals with biopsy-proven kidney disease in the training set [19]. Furthermore, NMR-based metabolomics was applied for the urinary metabolic profile and could provide a differential diagnosis of primary focal segmental glomerulosclerosis (FSGS) and other glomerulopathies [20]. In IgAN, metabolomic profiling has found that glycine levels increased without reduced eGFR compared to healthy, membranous nephropathy (MN), minimal change disease (MCD), or lupus nephritis (LN) [21]. However, integrative multi-omics analysis was rarely applied in IgAN. Thus, we performed a multi-omics study on IgAN, which could provide insights into various aspects.

This study employed a multi-omics workflow combining data-independent acquisition quantification proteomics and MS based untargeted metabolomics to identify the potential biomarkers for diagnosing IgAN from normal people. The plasma proteins and metabolic profiles of IgAN in the early stage have been characterized. After filtering the variables with the least absolute shrinkage and selection operator (LASSO) regression, potential biomarkers of IgAN were selected for constructing the diagnosis panel. Receiver operator characteristic (ROC) curve analysis exhibited high diagnostic sensitivity and specificity. Additionally, subsequent validation in an independent set demonstrated that the panel was a favorable diagnosis module. These analyses will inform strategies for the early diagnosis of IgAN.

## Methods and materials

### Study population

Samples of IgAN plasma were obtained from Zhejiang Provincial People’s Hospital at the time of diagnostic renal biopsies performed. Patients with a pathologically confirmed IgAN diagnosis and not treated with hormone immunosuppressants were included. People without kidney dysfunction constructed the healthy control group. Fifty-three individuals (33 patients with IgAN and 20 healthy controls) were enrolled for proteomic and metabolomic analyses. And 19 people (9 patients with IgAN and 10 healthy controls) were tested by ELISA in the validation set. The ethics committee approved this study for clinical studies at Zhejiang Provincial People’s Hospital. For plasma sampling, whole blood was collected in an EDTA tube and centrifugated at 1000 g for 10 min. Plasma was collected after centrifugation and kept at −80℃ until processed.

### Sample preparation for MS proteomic analysis

Samples of plasma were processed according to the previously described procedure with modifications [22]. Briefly, 100 μg of the protein was purified by methanol/chloroform precipitation. After centrifugation, the supernatant was removed, and the protein precipitate was dried. Afterward, the sample was resuspended in 50 mM NH_4_HCO_3_ solution and reduced with dithiothreitol (0.25 M in 50 mM NH_4_HCO_3_) for 30 min at 56 °C, then alkylated with 0.3 M chloroacetamide for 30 min at 37℃ in darkness. After alkylation, protein samples were diluted by adding 40 μL 100 mM NH_4_HCO_3_ and digested by trypsin at 37℃ for 16 h (enzyme to protein ratio of 1:100). The digestion was terminated by adding 20 μL 0.1% formic acid. After that, the samples were then purified using Ziptip (Merck Millipore, MA) C18 cartridges according to standard procedures. Mixed peptides for quality control were prepared by mixing equal volumes of each sample.

### Nanoflow liquid chromatography-tandem mass spectrometry analysis of proteomics

An Orbitrap 480 (Thermo Fisher Scientific, Dreieich, Germany) coupled with an EASY-nLC 1200 nanoflow liquid chromatography system (Thermo Fisher Scientific, Dreieich, Germany) was used to obtain mass spectral data. Then peptides were separated on an Acclaim Pep Map 100 C18 column (250 mm × 75 μm, 2 μm-C18) (Thermo Scientific Inc.). A binary mobile phase system was used, consisting of solvent A containing 0.1% formic acid in water and solvent B containing 80% acetonitrile in 0.1% formic acid/water at a flow rate of 300 nl/min. Elution for 65 min was performed by the following gradient: 0–1 min, 3%-5% B; 1–52 min, 8%—40% B; 52–56 min, 40%-95% B; 56–65 min, 95% B. Each peptide sample was ionized by an NSI source (Thermo Fisher Scientific, Dreieich, Germany). Data-independent acquisition (DIA) mode was used to detect the peptide ions. The spray voltage was set at static, and the ion transfer tube temperature to 320 °C. The full scan range was set to 325–1500 m/z, MS1 resolution to 120,000, MS1 automatic gain control (AGC targets) to 300, and MS1 maximum injection time to “Auto”. Fragmentation used higher‐energy collision disassociation (HCD) with a normalized collision energy of 30. The scan was equally divided into 48 continuous isolation windows for MS/MS scan, window overlap was 1 m/z. Precursors were prevented from being repeatedly sequenced by setting the expected peak width to 30 s. MS/MS resolution was set to 30,000, MS/MS AGC target to “Standard”, and MS/MS maximum injection time to “Auto”.

### Protein identification from the MS data

For the construction of the spectral library, the mixed peptides were fractionated and analyzed by LC–MS/MS in DDA mode. The DDA raw data were processed on the Protein Discoverer (Thermo Fisher Scientific, Dreieich, Germany) software with database UP000005640 downloaded from Uniprot. The result was imported into Skyline, and the spectral library was constructed. For protein quantification, chromatograms were extracted from RAW files acquired in DIA mode, and the target FASTA file was using database UP000005640. MSstats-Input file was exported and further processed using the R package MSstats for protein quantification and group comparison.

### Functional analysis of proteins

Gene set enrichment analysis (GSEA) was performed using the Kyoto Encyclopedia of Genes and Genomes (KEGG) subset of canonical pathways gene set in the Molecular Signatures Database (MSigDB). The KEGG database (http://www.genome.jp/kegg/) was used to map differentially expressed proteins (DEPs) pathway mapping. The clusterprofiler R package was used to annotate DEPs according to Gene Ontology (GO) database. A protein–protein interaction (PPI) network was constructed using the STRING database (http://string-db.org) and drawn in Cytoscape 3.9.0.

### Sample preparation for MS metabolomics analysis

The plasma sample (50 μL) was mixed with ketoprofen (internal standard, IS1) purchased by Makclin (Shanghai, China), Sulfamerazine (internal standard, IS2) purchased by Aladdin (Shanghai, China), 2-chloro-L-phenylalanine (internal standard, IS3) purchased by Makclin (Shanghai, China), and 150 μL of methanol (MS grade). The mixture was kept at − 20 ℃ for 30 min and centrifuged at 14,000 g at 4 ℃  for 20 min. The supernatant was further dried in a vacuum centrifugal concentrator. The quality control (QC) sample was prepared by mixing an equal aliquot from each plasma sample. All samples were redissolved by methanol in water (v:v = 2:1) before MS detection.

### Untargeted metabolomics assay

Ultra Performance Liquid Chromatography (UPLC) (Thermo Fisher Scientific, Dreieich, Germany) with Waters ACQUITY UPLC T3 C18 Column (2.1 mm × 100 mm, 1.8 um) was used to perform the separation at a flow rate of 0.30 mL/min. The injection volume was 8 μL, and the auto-sampler temperature was 4 °C. Solvent A (0.1% formic acid in water) and solvent B (0.1% formic acid in acetonitrile) were applied as mobile phases. Within 20 min, the analytes were separated in gradient condition: 0–1 min, 1% B; 1–15 min, 2% ~ 98% B; 15–18 min, 98% B; 18–20 min, 98% ~ 2% B. A polarity-switching mass spectrometer operated by Thermo Scientific Orbitrap 480 was used to detect metabolites with the spray voltage of 3.5 kV and -2.5 kV in positive and negative modes, respectively. Samples were ionized by electrospray ionization (ESI) (Thermo Fisher Scientific, Dreieich, Germany). The spray voltage was set at static, the ion transfer tube temperature to 300 °C, and the capillary temperature was 350 °C. Precursors were prevented from being repeatedly sequenced by setting the expected peak width to 15 s. The full scan range was set to 70–1050 m/z, MS1 resolution to 60,000, MS1 AGC targets to “Standard”, and MS1 maximum injection time to “Auto”. Fragmentation used HCD with a normalized collision energy of 30, 50, 150. MS/MS resolution was set to 15,000, MS/MS AGC target to “Standard”, and MS/MS maximum injection time to “Auto”.

### Metabolomics data processing

The acquired data were processed with Compound Discoverer 3.1 software (Thermo Fisher Scientific, Dreieich, Germany), including retention time correction, peak identification, peak extraction, peak integration, and peak alignment. Data were annotated by online databases KEGG, HMDB, mzCloud, and Chemspider. MetaboAnalyst (https://www.metaboanalyst.ca) was used to perform the principal component analysis. KEGG pathway databases and HMDB (https://hmdb.ca) were used to identify the top metabolic pathways associated with IgAN.

### Machine learning analysis

The LASSO regression analysis was performed in R using the glmnet package and binary logistic regression to construct the diagnosis model. To further assess the performance of the diagnosis model, a tenfold cross-validation was conducted. The ROC curve was used to evaluate the model diagnostic performance. And the diagnosis model was further verified by a support vector machine (SVM) and random forest.

### Validation of potential markers by ELISA assays

To further validate the reliability of the combination biomarkers, including PRKAR2A, IL6ST, SOS1, and palmitoleic acid, were selected for targeted analysis by ELISA assays. The independent batch of samples, including 9 plasma samples from the IgAN group and 10 from healthy people, were used for the assays. They were measured by a human PRKAR2A ELISA kit (PRKAR2A_AE1652A-96 T, Chuangshi, Tianjin, China), human Gp130/IL6ST ELISA Kit (EK0367, BOSTER, California, USA), human SOS1 ELISA (EH12498-96 T, FineTest, Wuhan, China) kit and human palmitoleic acid ELISA kit (CB15581-Hu, COIBO, Shanghai, China), respectively.

### Statistics analysis

Statistical analyses were conducted with the program R 4.1.0. Two-way unpaired Student's t-tests were used to compare two groups. Differences were considered significant at a p < 0.05.

## Result

### Study population

The research methodology comprised a preliminary finding driven by an integrated analysis of plasma proteomics and metabolomics of IgAN patients to identify the biomarkers and construct a diagnostic model externally validated by a validation cohort (Additional file 1: Figure S1). IgAN patients were diagnosed by biopsy, and those not treated with hormone immunosuppressants were selected. A total of 20 normal controls and 33 patients with IgAN were enrolled. A summary of the demographic and clinical characteristics of study participants was presented in Additional file 2: Table S1. The age in the IgAN group was 38.24 ± 13.16 years old, and the control group was 39.85 ± 14.56 years old. There was no significant difference in the distribution of age and gender in the two groups. In comparison, creatinine (CRE) and uric acid (UA) showed different levels in the two groups, which served as biochemical markers of renal damage.

### IgAN proteomic analysis and identification of DEPs

**Fig. 1:**
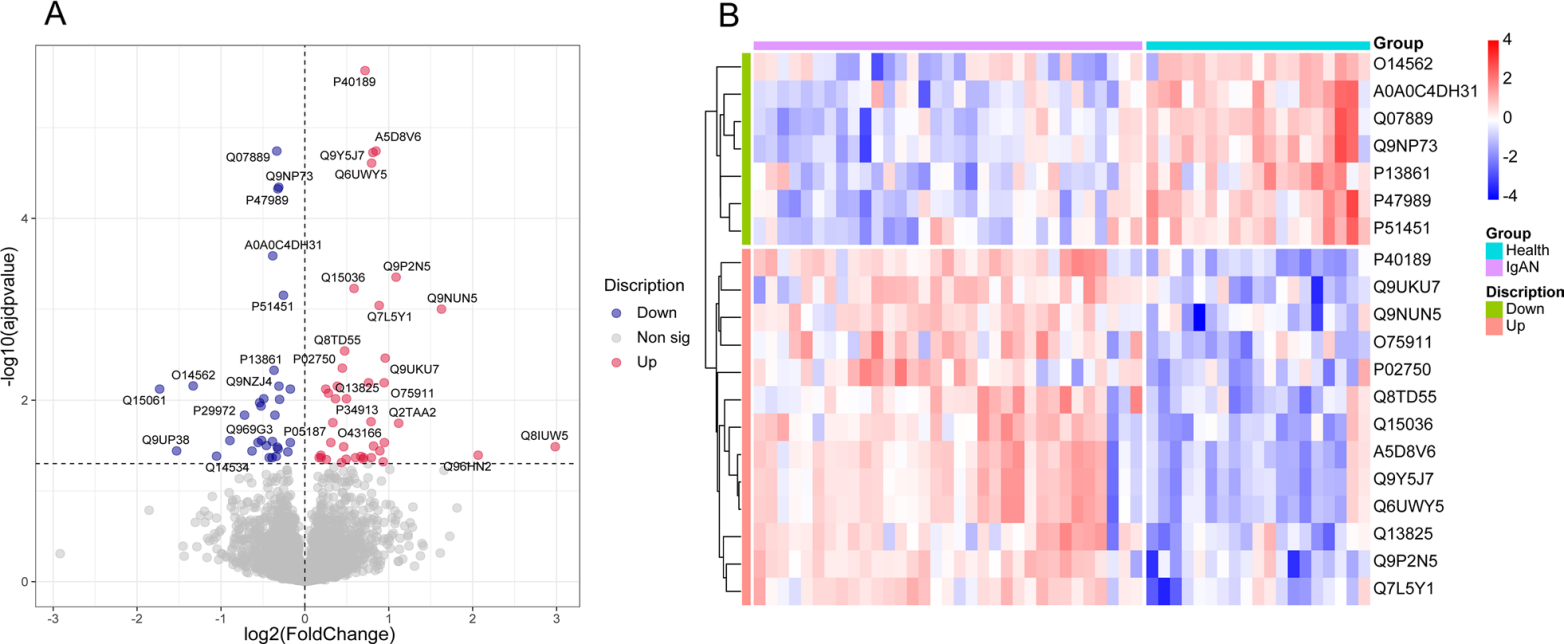
Proteomic analysis of IgAN. **A** The volcano plot showed DEPs between IgAN and health control. The red points represented 40 proteins that were up-regulated in the IgAN group (adjusted P < 0.05). The blue points represented 31 proteins that were down-regulated in the IgAN group (adjusted P value < 0.05) **B** The levels of DEPs in samples were analyzed by hierarchical clustering analysis. The heat map represented the Z scores of the top 20 DEPs in the IgAN group. (Red represents up-regulated, and blue represents down-regulated)

A total of 5267 proteins were identified. The quantified proteins are listed in Additional file 3: Table S2. Proteins with more than 50% missing values were discarded, and 5255 proteins remained. After normalizing the intensity values of each sample, the median of differences between samples can be normalized almost on the same line as found in the box plot (Additional file 1: Fig S2). Among the quantifiable proteins, 71 proteins exhibited a significant difference in IgAN compared to healthy controls (adjust p < 0.05, Additional file 2: Table S3). Among them, 40 proteins were up-regulated, and 31 were down-regulated, as demonstrated in Fig. 1A. Among the DEPs there were several proteins have been reported in the prediction of nephropathies, such as Immunoglobulin heavy variable 1–18 (IGHV1-18) [23], Beta-2-microglobulin (B2M) [24], Leucine-rich alpha-2-glycoprotein (LRG [25], and Aquaporin-1(APQ1) [26]. A hierarchical clustering algorithm was applied based on the top 20 DEPs (Fig. 1B). The result showed that the expression of DEPs could separate most IgAN samples from healthy controls.

### Functional enrichment analysis

**Fig. 2:**
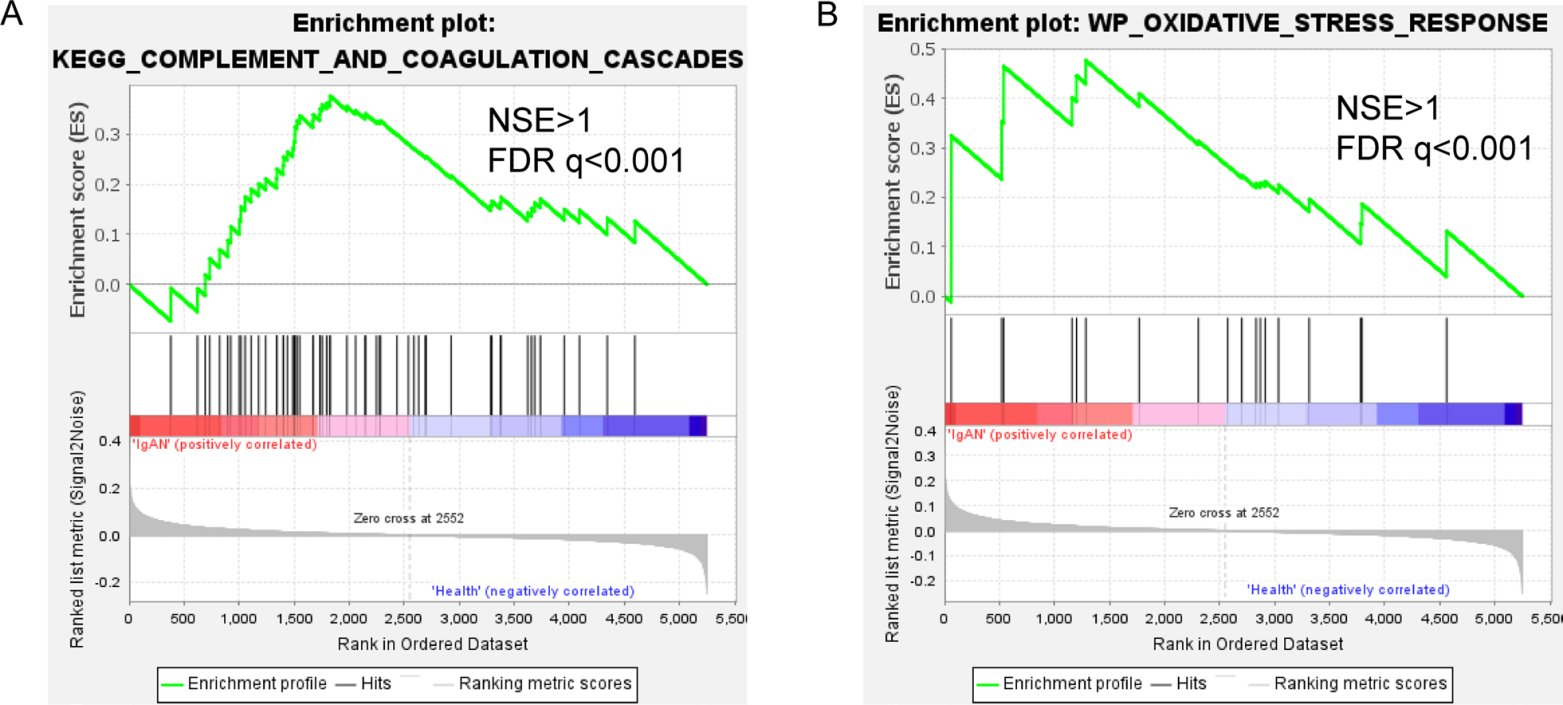
GSEA gene sets Enriched gene sets detected by GSEA in IgAN. **A**-**B** GSEA plots indicated significant enrichment of complement and coagulation cascades, and oxidative stress response in the IgAN compared with the healthy group. Genes were ordered according to their ranked ratios, and GSEA was performed using the GSEA (version 4.2.3) tool. The plot (green curve) shows the enrichment score (ES), for ranked genes compared with complement and coagulation cascades, and oxidative stress response gene set. The normalized enrichment scores (NESs) and false discovery rate (FDR) q-values (FDR q) were indicated

**Fig. 3:**
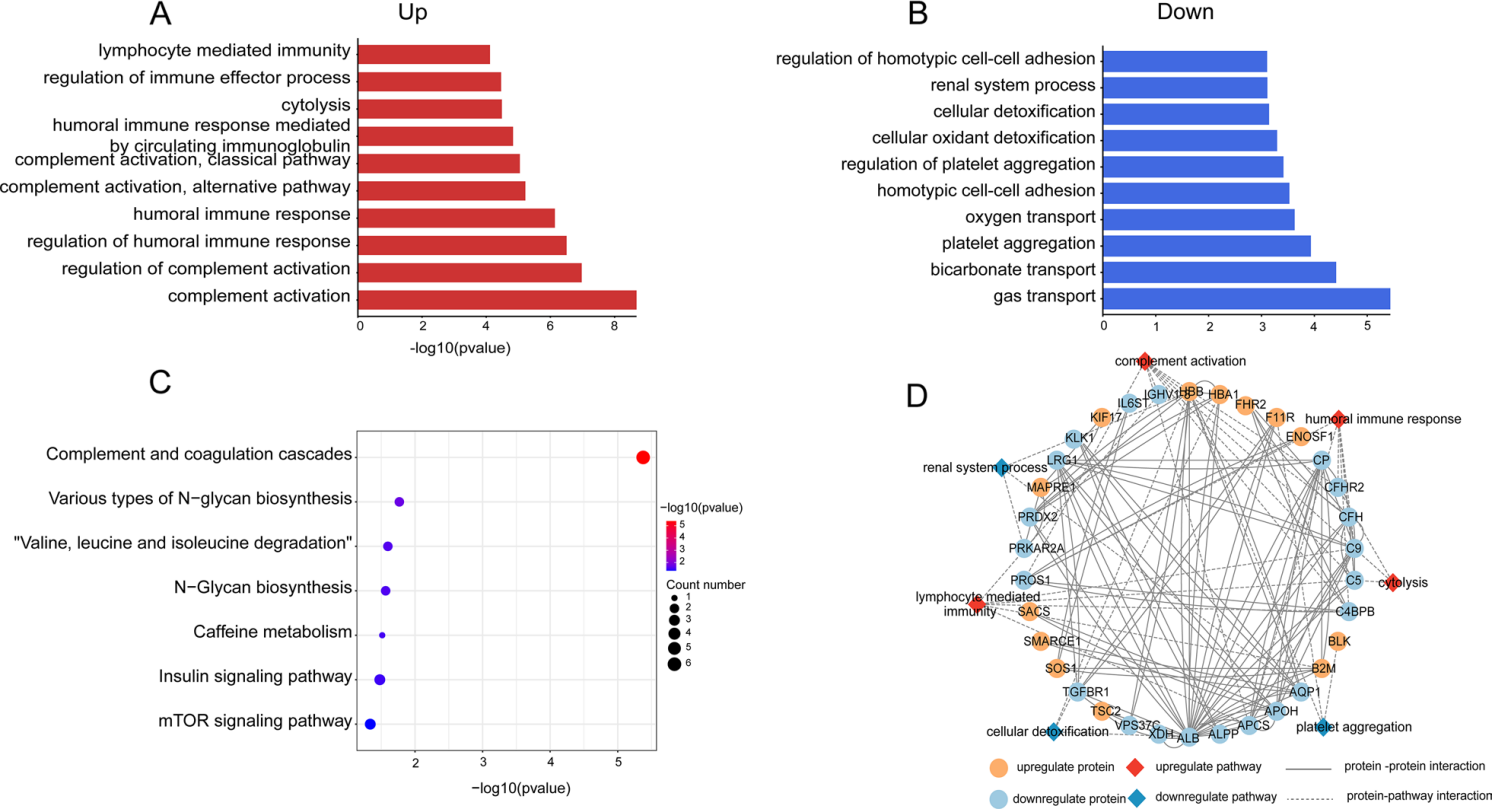
GO terms and KEGG pathway analysis for DEPs. **A** Up-regulated biological processes. GO annotation-biological processes enriched by up-regulated DEPs in IgAN. **B** Down-regulated biological processes. GO annotation-biological processes enriched by down-regulated DEPs in IgAN. **C** Enriched KEGG pathways of DEPs. **D** Proteins-pathways-proteins interaction network. Significantly altered biological process pathways were involved in the protein-protein interaction network of DEPs. GO, Gene Ontology; KEGG, Kyoto Encyclopedia of Genes and Genomes

GSEA predicted significantly altered signaling pathways in IgAN according to the normalized enrichment score (NES). Complement and coagulation cascades and MTOR signaling pathway were enriched in the IgAN subgroup (Fig. 2). Further, GO analysis was performed to identify the major functional categories of DEPs. Classification of the proteins of biological process terms showed that the increased proteins in IgAN patients were mainly associated with abnormal immune processes, including complement activation and regulation of humoral immune response. (Fig. 3A). The down-regulated proteins were primarily involved in platelet aggregation, renal system process, and regulation of homotypic cell-cell adhesion (Fig. 3B). Detailed information was provided in Additional file 2: Table S4. Next, KEGG pathway enrichment was performed. Complement and coagulation cascades, various types of N-glycan biosynthesis, and Valine and leucine, and isoleucine degradation were enriched (Fig. 3C). Next, with the STRING tool and Cytoscape, we analyzed the interaction network between these DEPs to understand their biological function and possible relationship better (Fig. 3D). These data demonstrated that several metabolic pathways were altered in IgAN patients, and the complement immune system is significantly activated among IgAN patients.

### IgAN metabolomic profile

**Fig. 4:**
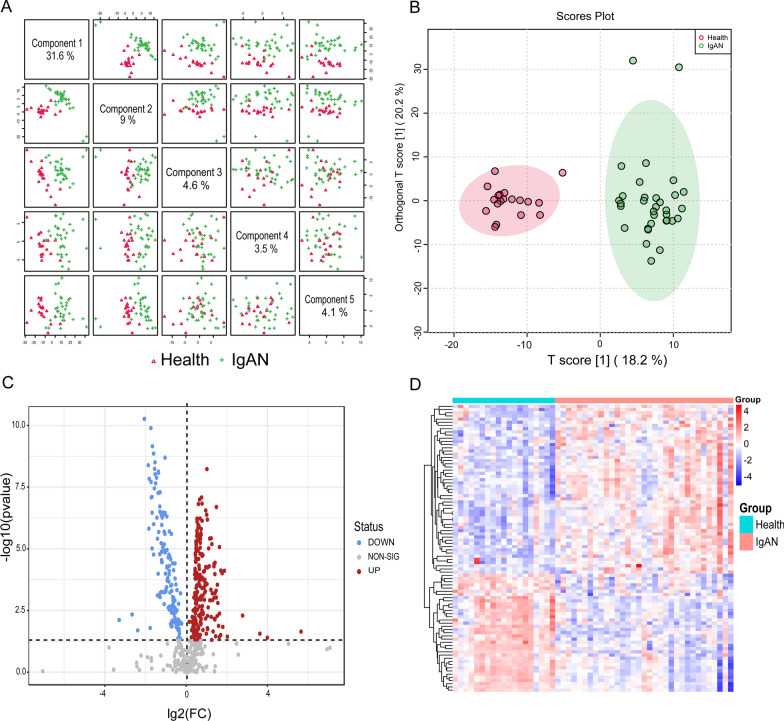
Multivariate data analysis of IgAN metabolomics data. **A** Principal Component (PC) scores plot from PLS-DA model. The diagonal shown the pairwise scoring plots between the five principal components with the corresponding variances. **B** OPLS-DA score plot showed the separation between the control group and IgAN. **C** Volcano plot of DEMs. The red points represented up-regulated metabolites, and the blue points characterized down-regulated metabolites in the IgAN group. **D** Heat map presenting DEMs between the IgAN and control groups. (Red represents up-regulated, and blue represents down-regulated in the IgAN group)

Untargeted metabolomics was analyzed to grasp the change of metabolites in IgAN, and 540 metabolites were identified finally. The metabolite data of IgAN and controls were used for unsupervised and supervised pattern recognition analysis to investigate the potential of metabolite profiling in IgAN diagnosis. The principal component analysis (PCA) results showed that IgAN plasma samples were clustered together, but there was some overlap with healthy controls (Figure S3). PLS-DA showed that PC1, PC2, and PC3 explained 32.7%, 9%, and 6.3% of the difference in the two groups (Fig. 4A). A clear separation between IgAN patients versus healthy controls was observed on the OPLS-DA score plot (Fig. 4B).

### DAMs between healthy controls and IgAN patients

The metabolites with variable importance in projection (VIP) values greater than 1.0 and p < 0.05 were considered as DAMs. 90 out of 540 metabolites were identified (Fig. 4C). 53 increased DAMs and 37 decreased DAMs were identified in the IgAN group (Fig. 4D). All the DAMs were annotated and classified into nine categories in the HMDB database. Information about the metabolites was listed in Additional file 2: Table S5.

**Fig. 5:**
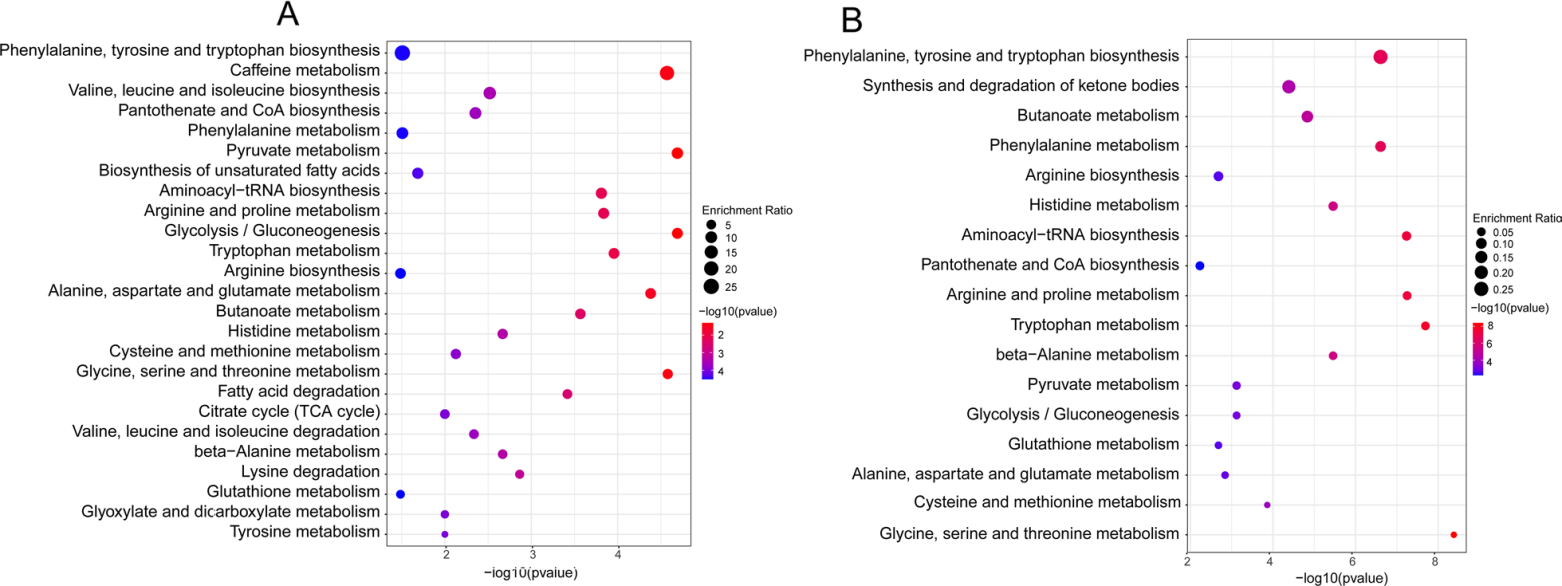
Enrichment analysis of metabolites using Metabolanalyst 5.0 online software. **A** The metabolome set enrichment analysis (MSEA) for identifying significantly altered metabolic pathways. **B** KEGG pathway enrichment analysis of DAMs. (P < 0.05; VIP-Scores > 1)

All annotation metabolites were then imported into MetaboAnalyst 5.0 for pathway analysis. The metabolome set enrichment analysis (MSEA) showed that glycolysis/gluconeogenesis, pyruvate metabolism, and amino acid metabolism were changed in IgAN. (Fig. 5A). And DAMs were mapped to the KEGG pathway to explore the metabolic alterations (Fig. 5B). The result showed that 17 metabolite pathways were changed, including carbohydrate, energy, and amino acid metabolism. A summary of the altered abundances of these pathways and metabolites was exhibited in Additional file 2: Table S6. And a heatmap of the DAMs mapped on KEGG pathways was illustrated (Additional file 1: Figure S4).

### Construction of the diagnostic model for IgAN

**Fig. 6:**
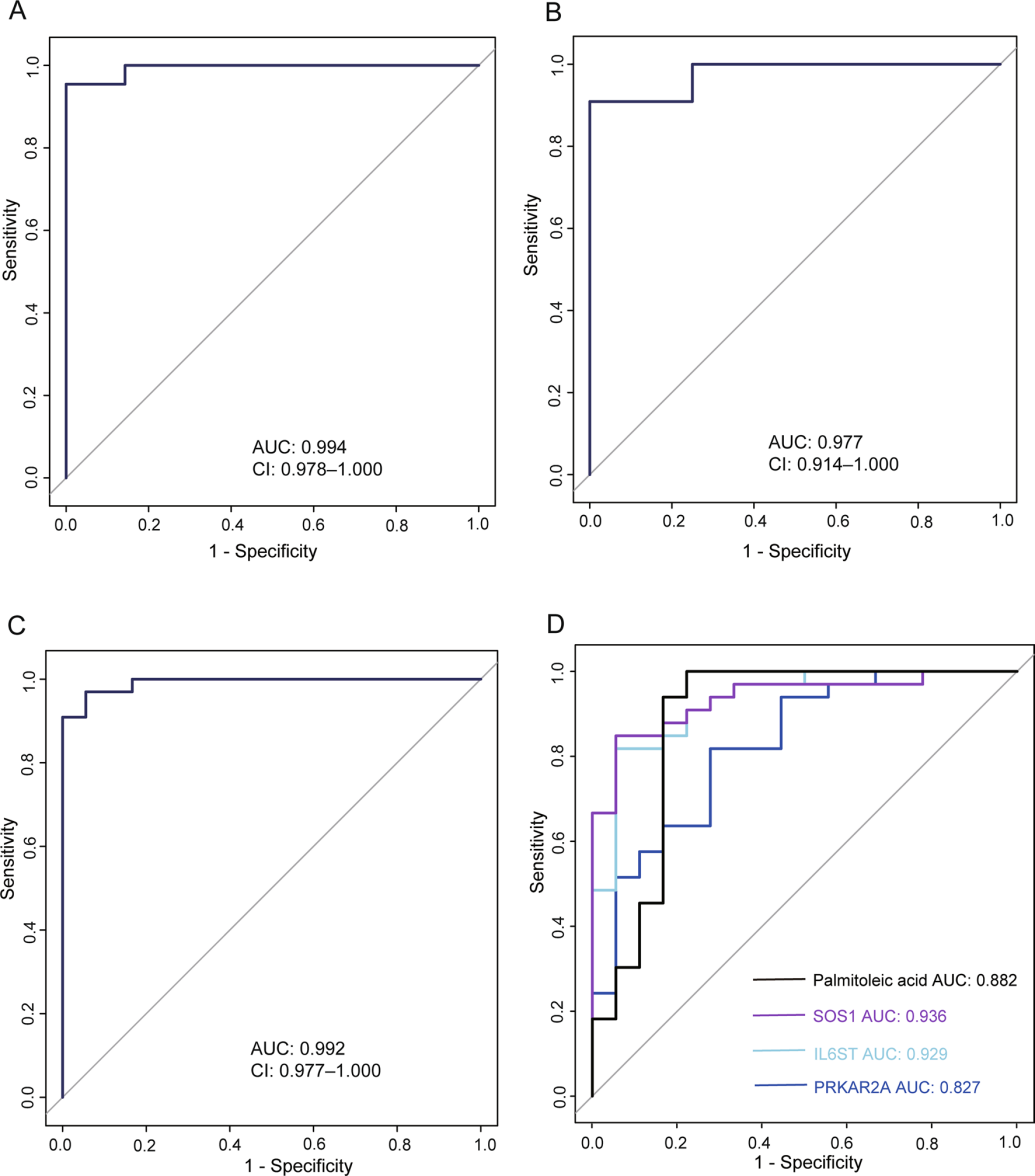
The diagnostic efficacy of biomarkers in IgAN. **A** ROC analysis of the combined model was performed to evaluate the diagnostic performance in the training set. **B** ROC analysis of the combined model was performed to assess the diagnostic performance in the testing set. **C** ROC analysis of the combined model was performed to evaluate the diagnostic performance in the entire collection. **D** ROC analysis of the four biomarkers was performed to evaluate the diagnostic performance in the entire collection, respectively

Potential biomarkers of IgAN were then identified via comprehensive proteomic and metabolomic profiling. Exposomes and vitamin metabolites were excluded because vitamin supplements, medications, and dietary factors may affect metabolite levels independently of disease status. The DEPs and the rest of the DAMs were used for machine learning to build classification models that could distinguish IgAN from normal people. To verify the accuracy of the signature, the whole dataset was divided into the training set and the testing set in a ratio of 7:3. The LASSO regression model was used to determine the diagnostic markers of IgAN in the training set. Three unique proteins and one metabolite were identified as potential biomarkers, including PRKAR2A (P13861), IL6ST (P40189), SOS1 (Q07889), and Palmitoleic acid. A ROC analysis was applied after calculating the predicted probabilities to evaluate the diagnostic accuracy of the four selected biomarkers. The AUC of the combination of these four biomarkers was 0.994 (Fig. 6A). In the test set, the four biomarkers also showed good diagnostic performance, and the AUC was 0.977 (Fig. 6B). Meanwhile, the four biomarkers can predict IgAN with high precision in the whole set, and the AUC was 0.992 (Fig. 6C). The algorithm achieved an accuracy of 97.2% (recall = 0.955, precision = 1) in the training set, 93.3% in the test set (recall = 0.909, precision = 1), and 96.1% in the whole set (recall = 0.970, precision = 0.970). Next, we analyzed the levels of the biomarkers in all samples. There were significant changes in four biomarkers between IgAN and health control. The level of IL6ST was increased, while the levels of PRKAR2A, SOS1, and palmitoleic acid were decreased in the IgAN group. The levels of the four biomarkers were displayed in Figure S5. Based on logistic regression analysis, the four biomarkers yielded a diagnosis of IgAN, respectively, with a ROC > 0.8 in the whole set (Fig. 6D). For each analyte and the combination of biomarkers, the accuracy, recall (sensitivity or true positive rate), precision, and AUC were present in Table1. In short, these results showed that the combination of biomarkers significantly increased the predictive ability of the model. Additionally, random forest classification and SVM were used to further validate the model retrieved from the cross-validation procedure. Supervised random forest classification using the four metabolites had a classification accuracy of 100%, and supervised SVM also showed a 100% accuracy (Table 2).

**Table 1:** The table reports the accuracy, recall, precision, and AUC measures for each individual marker and the combination

	Accuracy	Recall	Precision	AUC
PRKAR2A	0.784	0.818	0.844	0.827
IL6ST	0.863	0.818	0.964	0.929
SOS1	0.882	0.849	0.966	0.936
Palmitoleic acid	0.922	1	0.892	0.882
Combination	0.980	0.970	1	0.997

**Table 2:** Classification table of random forest method and SVM

Random forest classification, accuracy = 100%, N = 51		
	Health	IgAN
Health	18	0
IgAN	0	33

Support vector machine classification, accuracy = 100%,N = 51		
	Health	IgAN
Health	18	0
IgAN	0	33

### External validation of biomarkers

**Fig. 7:**
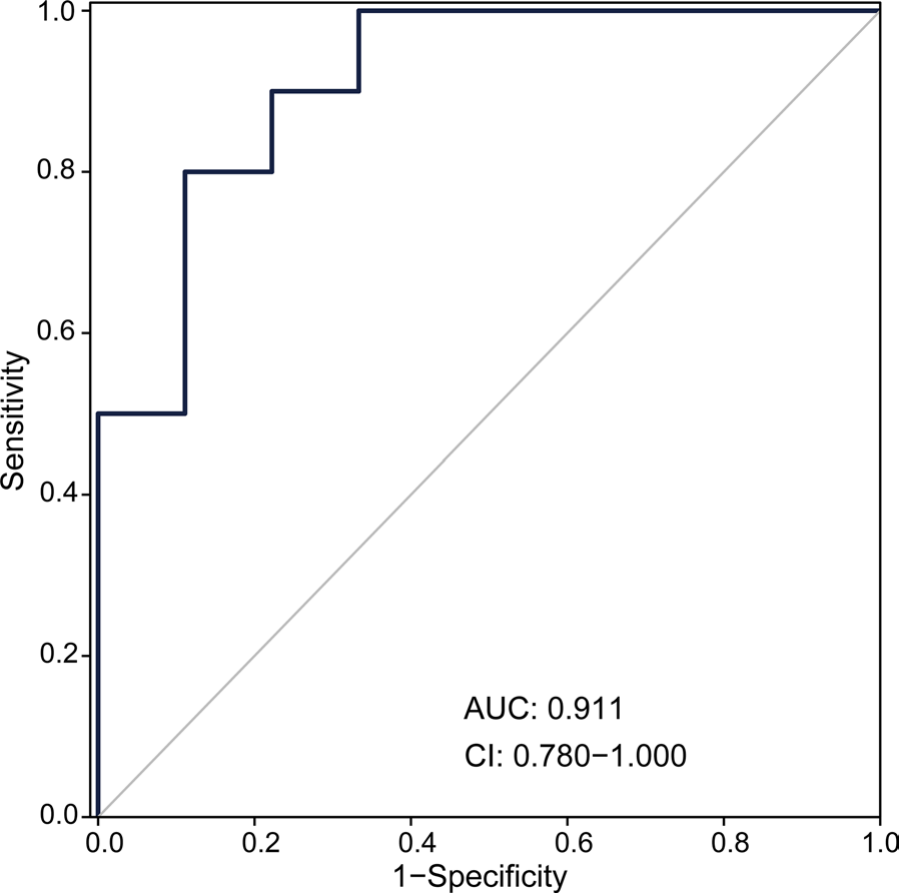
ROC analysis in the validation set. The performance of the combined diagnostic model in the validation set was evaluated by ROC analysis

An independent cohort of 10 healthy controls and 9 patients with IgAN was used for external validation of the four biomarkers. Demographic and clinical data of the study group was presented in Additional file 2: Table S7. ELISA assays determined the content of the four biomarkers. The ROC curve showed that the diagnostic ability of the four biomarkers was 0.911 (Fig. 7). The result further proved that these three proteins and a metabolite were potential biomarkers for separating IgAN from health controls.

## Discussion

IgAN was defined as a common type of primary GN [27], one of the leading causes of ESRD [14]. The diagnosis was primarily based on changes in kidney pathology, and patients have heterogeneous characteristics and different prognoses. Though aberrant glycosylation [28], circulating immune complex deposition [29], and immune cell infiltration [30] were reported to be involved in the IgAN, the pathogenesis of IgAN has not been fully elucidated. Identifying the involved pathways and these critical proteins and metabolites affected by IgAN using modern MS technological means is useful to augment our understanding of IgAN. This study integrating proteomics and metabolomics approached found that various pathways were disturbed in the early stage of IgAN.

### Proteins associated with complement in IgAN plasma

Blood protein analysis is an important clinical application of proteomics, but its dynamic range presents a challenge. Proteome in-depth analysis is necessary for identifying low-abundance proteins in experiments. So DIA mode was used to perform proteomic analysis on samples [31]. In this study, 71 DEPs were identified in the IgAN by DIA proteomic analysis. These proteins play a critical role in complement activation and humoral immune response. Complement activation has apparent alterations in IgAN patients. Excessive complement activation may be involved in a crescent formation, predominantly diffuse crescent formation [32]. We found that complement-associated proteins have been activated in early-stage IgAN patients, even though the value of eGFR was normal.

### Amino acid metabolism of DAMs in IgAN plasma

Between IgAN patients and healthy individuals, we detected a series of dysregulated amino acid metabolism. Among them, tryptophan, kynurenine, and ornithine were increased. Metabolic disturbances of amino acids were also observed in another IgAN metabolomics study, in which alanine, glycine, and isoleucine levers were significantly up-regulated [21]. Besides, tryptophan was an essential amino acid for an organism’s energy metabolism and was a precursor material for 5-hydroxytryptamine and kynurenine [33]. The kynurenic acid pathway was thought to be the main catabolic route for tryptophan [34]. The metabolism of tryptophan was related to the production of urine toxin in chronic kidney disease and impaired renal function in diabetic nephropathy, which promotes oxidation, inflammation, coagulation, and apoptosis [35, 36]. Metabolomics results showed abnormal tryptophan metabolism in IgAN patients, with significantly elevated levels of 5-hydroxyindoleacetate and kynurenine. Therefore, amino acid metabolism dysregulation also existed in IgAN patients and influenced the process of the disease.

### Biological functions of biomarkers

Considering the clinical feasibility for early diagnosis of IgAN, a concise multi-omic classifier containing PRKAR2A, IL6ST, SOS1, and palmitoleic acid was created. PRKAR2A is the protein kinase cAMP-dependent type II regulatory subunit alpha and has been reported to play a core role in regulating neuroinflammatory processes [37]. Several studies suggested that PRKAR2A was associated with inflammatory pain and that antagonizing PRKAR2A offered a practical solution to controlling inflammatory pain [38, 39]. A systematic literature review of IgAN has described that across 8 studies reporting health-related quality of life, pain and fatigue were the most reported symptoms [40]. In this study, decreased abundance of plasma PRKAR2 was observed among IgAN patients. The results indicated that the variation of PRKAR2A may play a role in the pain symptoms of IgAN, and it can be further studied as a biomarker for regular screening of IgAN patients. IL6ST, also names gp130, is the signaling subunit of receptors for cytokines of the IL-6 family [41] and plays a crucial role in maintaining immunity, mediating immune responses, and regulating inflammation [42]. In vivo, selective inhibition of trans signal transduction by a fusion protein containing two soluble forms of gp130 (sgp130) chains linked by an inactivated Fc receptor portion (sgp130Fc) alleviated disease progression in rapidly progressive glomerulonephritis mice [43]. In vitro studies revealed that inhibition of IL6ST expression could reduce the level of fibrosis-related genes in HG-treated cells [44]. According to the present research results, IL6ST may involve in the activation of inflammatory pathways and contribute to the progression of IgAN nephropathy. This research showed that IL6ST can distinguish IgAN from health and can be biomarkers for further study. SOS-1 is a guanine nucleotide exchange factor for RAS proteins. The expression of SOS1 can affect intracellular ROS levels, and the loss of SOS1 leads to specific changes in mitochondrial shape, mass, and dynamics, resulting in energy metabolism disorders [45]. A previous study showed that Ras-Sos-1 played a significant role in regulating renal tubular epithelial-to-mesenchymal transition [46]. Renal tubule epithelial mesenchymal transformation is one of the main causes of renal fibrosis reflected the clinical severity of IgA nephropathy [47]. This study found that the expression of SOS-1 was significantly different between IgAN and normal people. SOS-1 may be involved in the epithelial mesenchymal transformation to a large extent in IgAN. Additionally, metabolomic data suggested dysregulation of energy metabolism in IgAN patients indicating SOS-1 may thus be a robust diagnostic biomarker for IgAN. Palmitoleic acid is an omega-7 monounsaturated fatty acid (MUFA) [48]. Incubation with palmitoleate can reverse the proinflammatory gene expression and cytokine secretion seen in bone marrow-derived macrophages from high-fat fed mice [49]. Supplementation with palmitoleic acid or palmitoleic acid enriched oils attenuated hypertrophy of adipocytes and inflammation in the adipose tissue and macrophages [50]. A clinical study found that patients with end-stage renal disease have lower palmitoleic acid levels than the control group [51]. In the present study, the level of palmitoleic acid was lower IgAN patients than normal. We speculate that palmitic acid may also play an anti-inflammatory role in IgAN nephropathy, and supplementation of palmitic acid may reduce the inflammatory response of IgAN. But more experiments are needed to confirm this hypothesis. Additionally, the above four markers selected by LASSO logistic regression showed that the combination of markers was more reliable than a single marker for diagnosing IgAN. Further analysis of the ROC curve results exhibited that the combined markers produced the highest AUC of 0.992 in the whole discovery set, which was significantly higher than every single marker.

To sum up, LC-MS/MS based proteomics and metabolomics were performed to analyze the IgAN plasma protein and metabolic profiling and identify the potential biomarkers. A combination of multi-omic biomarkers might provide more reliable information for early diagnosis. In addition, the age and sex of the research cohort were matched for the omics analysis, which would minimize the interference of objective factors. Moreover, these candidate biomarkers might be potential molecular targets for drug discovery and improve the early-IgAN diagnosis. However, there are still some limitations to this study. The number of samples was limited, and large cohorts are needed to further validate the performance of the diagnostic model. Plasma from patients of other nephropathy is also needed to evaluate the sensitivity and specificity of the diagnostic model. And though we have found that the complement and the immune system were activated and the energy and amino acid metabolism were disordered in the IgAN patients, the exact pathogenesis of IgAN remains to be further studied.

## Conclusion

In summary, integrative DIA proteomics and MS based untargeted metabolomics analyses of plasma were performed to discover the IgAN diagnosis biomarkers panels. Proteomics and metabolomics analyses of IgAN plasma revealed that the complement and the immune system were activated, while the energy and amino acid metabolism were disordered in IgAN patients. The biomarker panels containing PRKAR2A, IL6ST, SOS1, and palmitoleic acid, were selected by LASSO, which can be used as early warning and diagnosis of IgAN.

## Supplementary Information

**Additional file 1: Figure S1.** An analysis of multi-omics comparison overview workflow. Plasma was collected from IgAN and the healthy population for proteomic and metabolomic analysis. Protein and metabolism profile analysis utilizing UPLC-MS/MS was used to identify systematically. Bioinformatics analyses were used to screen potential biomarkers in IgAN. **Figure S2.** Logarithmic transformation was used to normalize the proteomic data. The boxplots showed that the distribution of values for each sample was consistent after normalization. **Figure S3.** Principal Component Analysis (PCA) analysis of the IgAN and health control samples based on metabolomics. Each data point corresponds to the PCA analysis of each sample. **Figure S4.** The heatmap illustrates clustering by unsupervised hierarchical clustering with euclidean distance based on differentially expressed metabolites mapped in metabolic pathways. **Figure S5.** Mass spectrometry-based relative quantifications of four biomarkers expression in IgAN and health. (A-D) Boxplots showed the relative intensities of the four biomarkers in plasma, including PRKAR2A (P13861), IL6ST (P40189), SOS1(Q07889), and palmitoleic acid between IgAN and health control. (＊＊＊ indicates P <0.001).

**Additional file 2: Table S1.** Clinical characteristics of the patients with IgAN and normal control in the discovery cohort. **Table S3.** The expression of DEPs in IgAN. **Table S4. **The top 10 GO BP analysis of upregulate and downregulate in DEPs of IgAN. **Table S5.** The expression of DAMs in IgAN. **Table S6.** The pathway of DAMs enrichment in IgAN. **Table S7.** Clinical characteristics of the patients with IgAN and normal control in the validation cohort.

**Additional file 3: Table S2. **The quantified proteins in IgAN and health.

## Data Availability

All data generated or analyzed during this study are included in this published article.

## Abbreviations

AUC: Area under the curve
CKD: Chronic kidney disease
DAMs: Differentially abundant metabolites
DEPs: Differentially expressed proteins
DIA: Data-independent acquisition
ESRD: End-stage renal disease
FSGS: Focal segmental glomerulosclerosis
GN: Glomerulonephritis
IgAN: Immunoglobulin A nephropathy
LASSO: Least absolute shrinkage and selection operator
LC-MS/MS: Liquid chromatography-tandem mass spectrometry
LN: Lupus nephritis
