# Supplementary material for: LC-MS/MS based metabolomics and proteomics reveal candidate biomarkers and molecular mechanism of early IgA nephropathy
Source: Clin Proteomics. 2022 Dec 27;19:51. doi: 10.1186/s12014-022-09387-5 (PMC9793667; doi:10.1186/s12014-022-09387-5)
Supplement: Supplementary file 1 — Additional file 1: Figure S1. An analysis of multi-omics comparison overview workflow. Plasma was collected from IgAN and the healthy population for proteomic and metabolomic analysis. Protein and metabolism profile analysis utilizing UPLC-MS/MS was used to identify systematically. Bioinformatics analyses were used to screen potential biomarkers in IgAN. Figure S2. Logarithmic transformation was used to normalize the proteomic data. The boxplots showed that the distribution of values for each sample was consistent after normalization. Figure S3. Principal Component Analysis (PCA) analysis of the IgAN and health control samples based on metabolomics. Each data point corresponds to the PCA analysis of each sample. Figure S4. The heatmap illustrates clustering by unsupervised hierarchical clustering with euclidean distance based on differentially expressed metabolites mapped in metabolic pathways. Figure S5. Mass spectrometry-based relative quantifications of four biomarkers expression in IgAN and health. (A-D) Boxplots showed the relative intensities of the four biomarkers in plasma, including PRKAR2A (P13861), IL6ST (P40189), SOS1(Q07889), and palmitoleic acid between IgAN and health control. (＊＊＊ indicates P <0.001). [file 12014_2022_9387_MOESM1_ESM.doc]

**LC-MS/MS based Metabolomics and Proteomics Reveal Candidate Biomarkers and Molecular Mechanism of Early IgA Nephropathy**
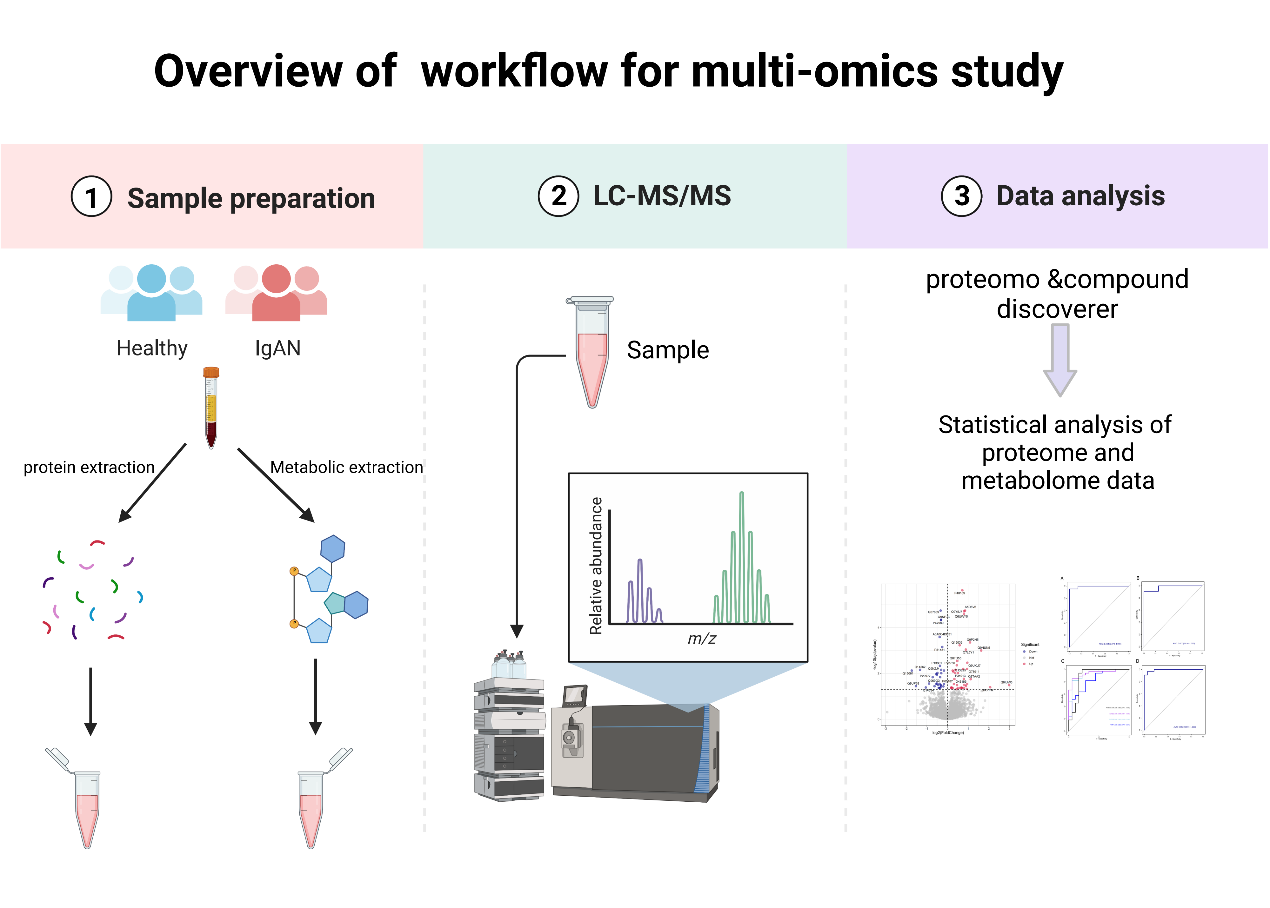


Figure S1 An analysis of multi-omics comparison overview workflow. Plasma was collected from IgAN and the healthy population for proteomic and metabolomic analysis. Protein and metabolism profile analysis utilizing UPLC-MS/MS was used to identify systematically. Bioinformatics analyses were used to screen potential biomarkers in IgAN.


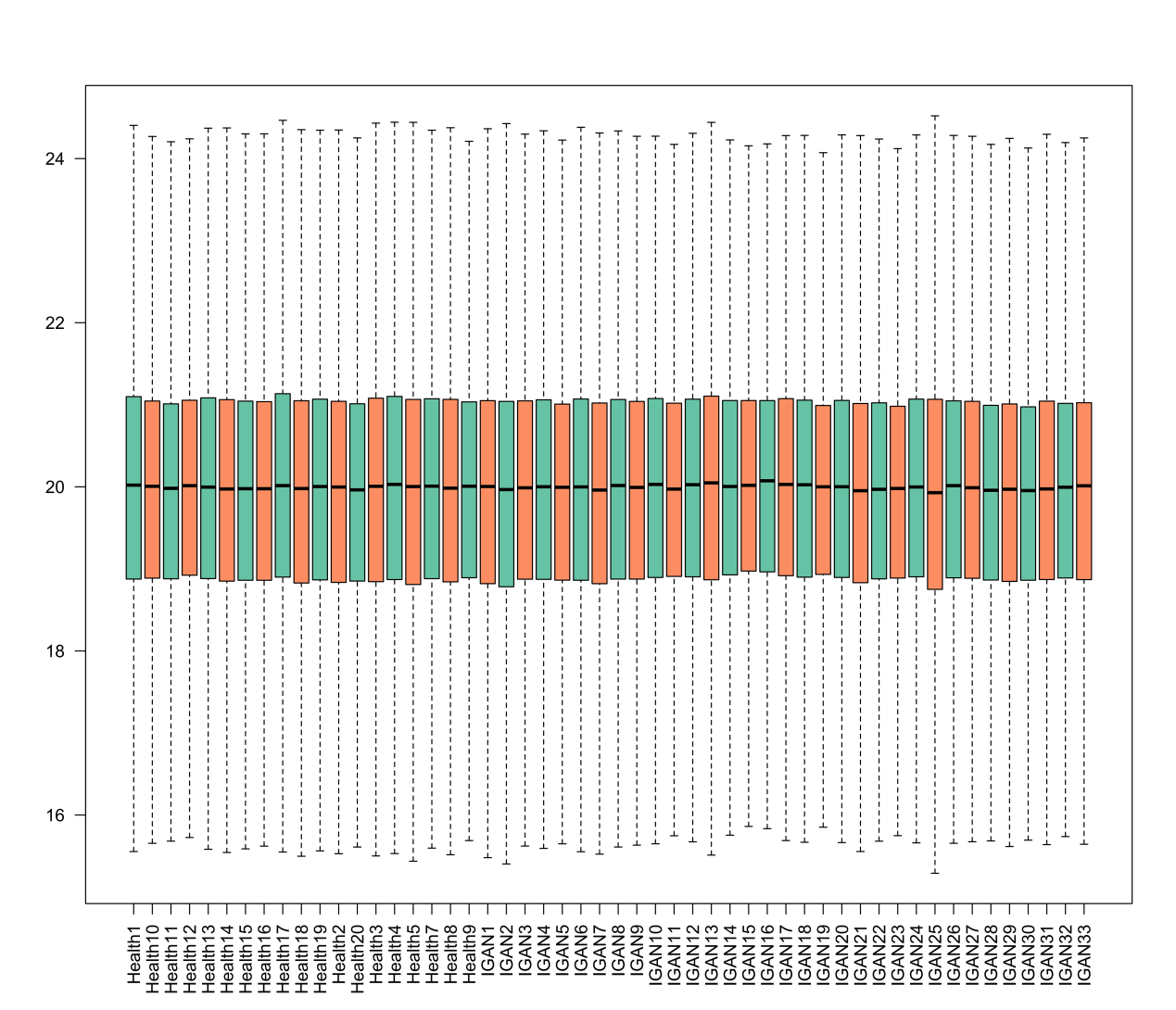


Figure S2 Logarithmic transformation was used to normalize the proteomic data. The boxplots showed that the distribution of values for each sample was consistent after normalization.


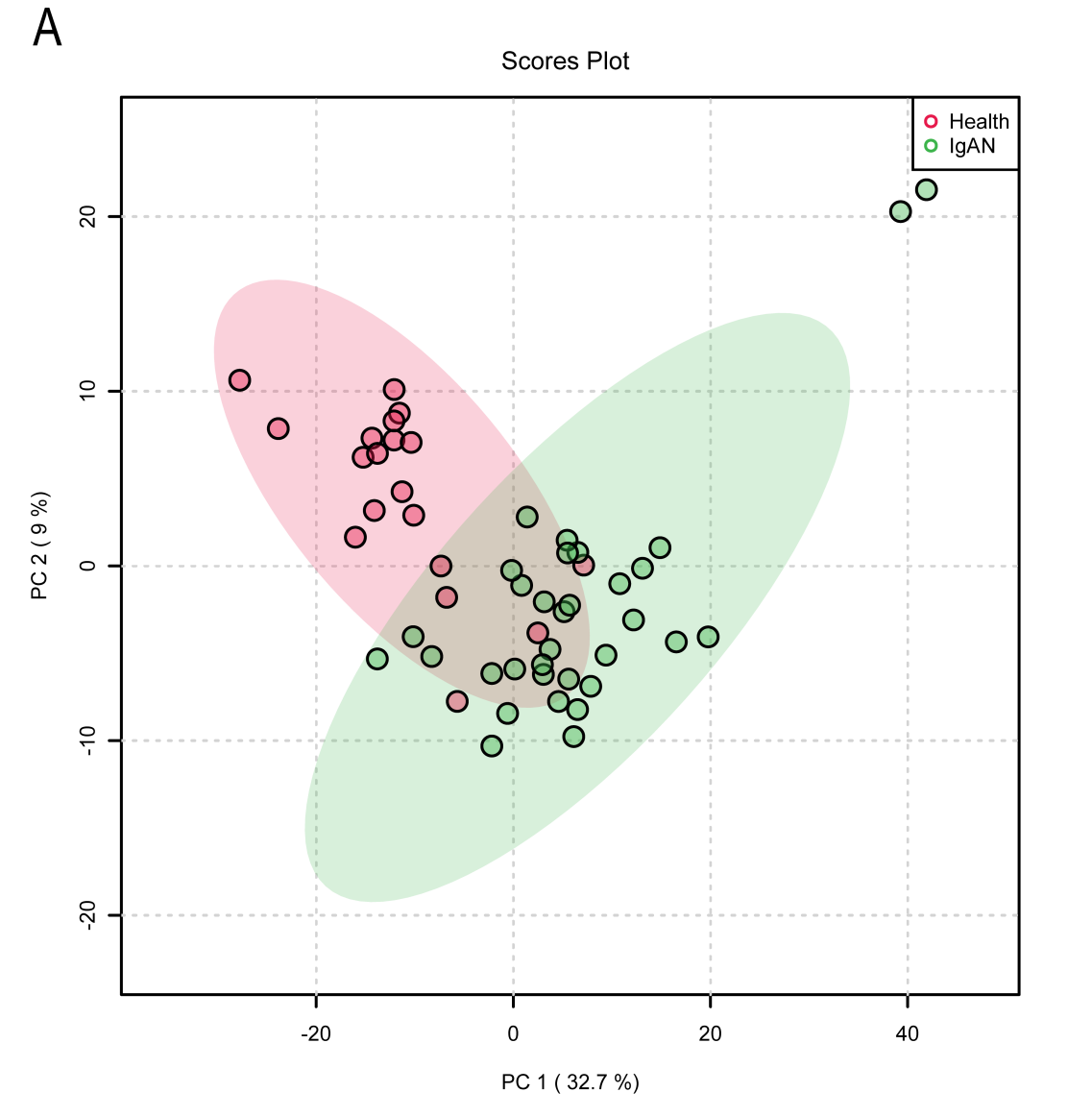


Figure S3 Principal Component Analysis (PCA) analysis of the IgAN and health control samples based on metabolomics. Each data point corresponds to the PCA analysis of each sample.


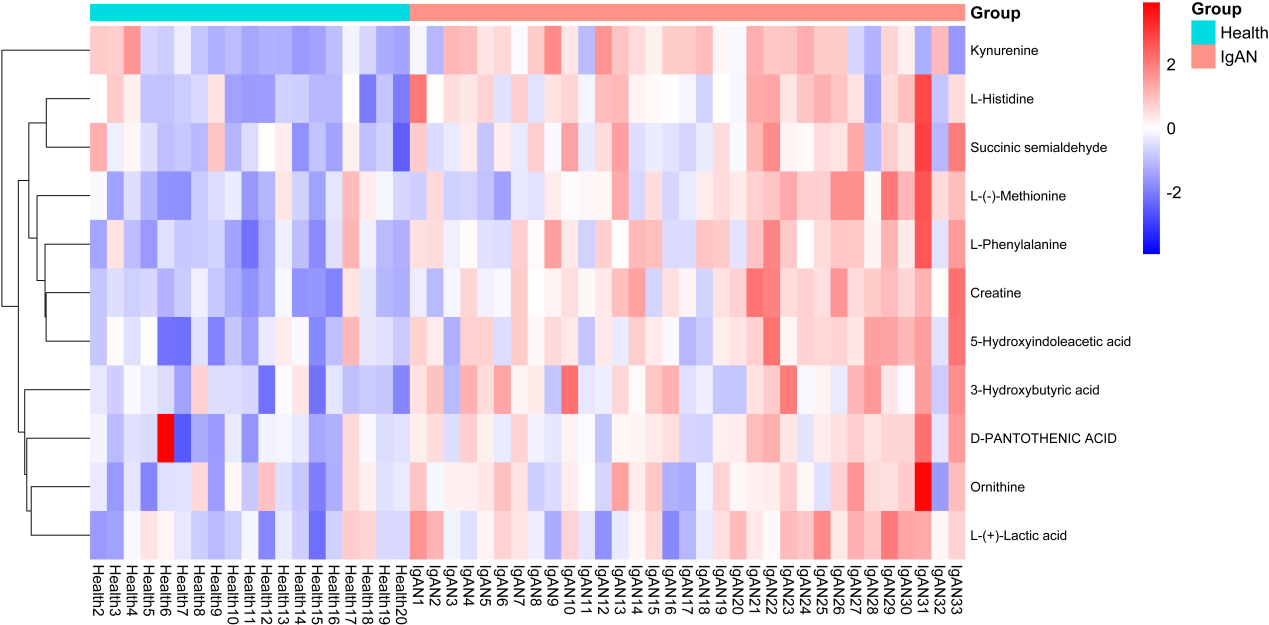


Figure S4 The heatmap illustrates clustering by unsupervised hierarchical clustering with euclidean distance based on differentially expressed metabolites mapped in metabolic pathways.


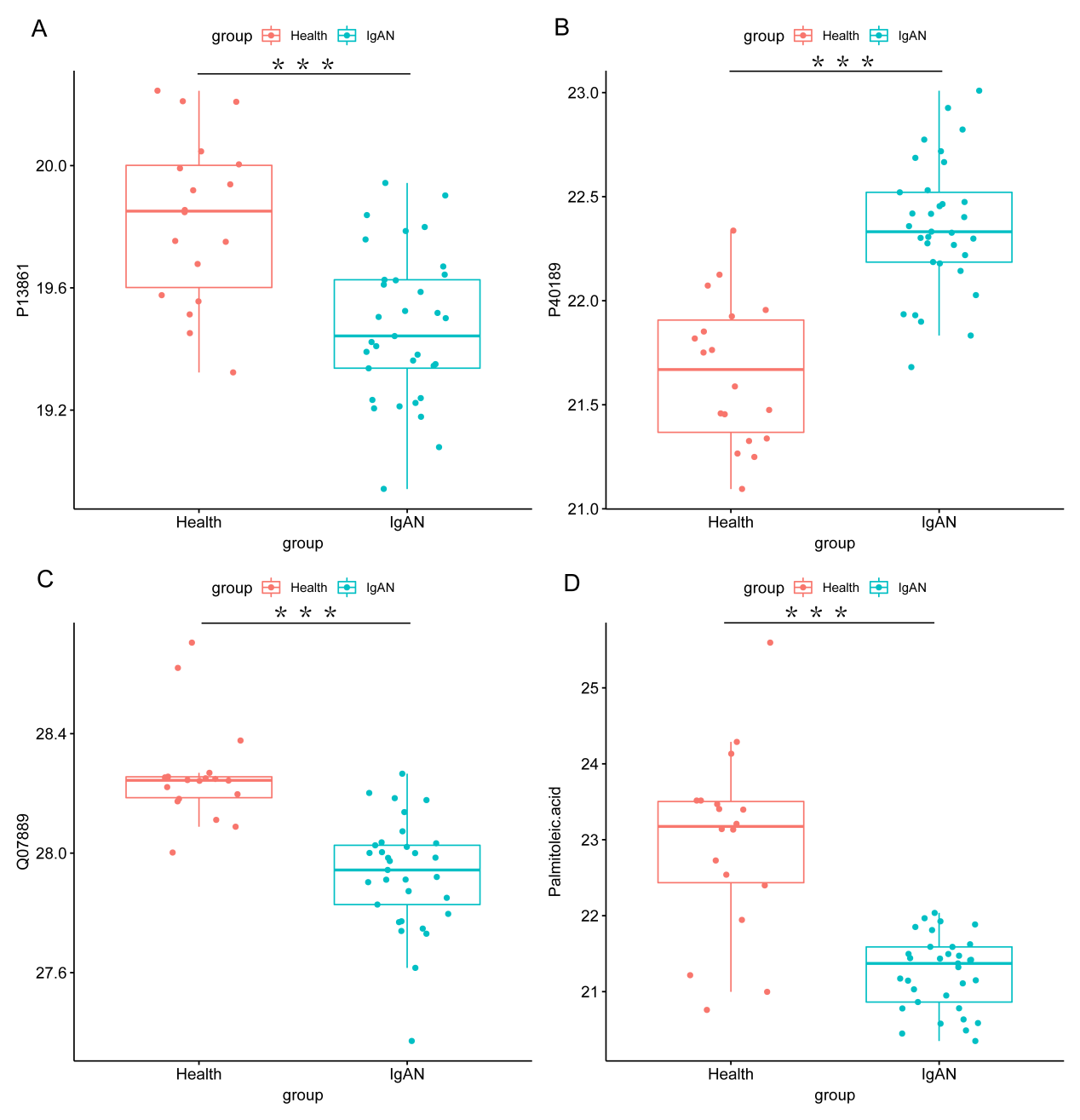


Figure S5 Mass spectrometry-based relative quantifications of four biomarkers expression in IgAN and health. (A-D) Boxplots showed the relative intensities of the four biomarkers in plasma, including PRKAR2A (P13861), IL6ST (P40189), SOS1(Q07889), and palmitoleic acid between IgAN and health control. (＊＊＊ indicates P <0.001)
