# Supplementary material for: LC-MS/MS based metabolomics and proteomics reveal candidate biomarkers and molecular mechanism of early IgA nephropathy
Source: Clin Proteomics. 2022 Dec 27;19:51. doi: 10.1186/s12014-022-09387-5 (PMC9793667; doi:10.1186/s12014-022-09387-5)
Supplement: Supplementary file 2 — Additional file 2: Table S1. Clinical characteristics of the patients with IgAN and normal control in the discovery cohort. Table S3. The expression of DEPs in IgAN. Table S4. The top 10 GO BP analysis of upregulate and downregulate in DEPs of IgAN. Table S5. The expression of DAMs in IgAN. Table S6. The pathway of DAMs enrichment in IgAN. Table S7. Clinical characteristics of the patients with IgAN and normal control in the validation cohort. [file 12014_2022_9387_MOESM2_ESM.doc]

Additional file 2: Table S1. Clinical characteristics of the patients with IgAN and normal control in the discovery cohort.

| Characteristic | Health | IgAN | p |
| --- | --- | --- | --- |
| n | 20 | 33 |  |
| Gender, n (%) |  |  | 1.000 |
| female | 10 (18.9%) | 17 (32.1%) |  |
| male | 10 (18.9%) | 16 (30.2%) |  |
| Age, mean ± SD | 39.85 ± 14.56 | 38.24 ± 13.16 | 0.681 |
| CRE, median (IQR) | 56.84 (48.41, 64.93) | 85.5 (71.8, 106.2) | < 0.001 |
| UA, median (IQR) | 294.91 (260.24, 324) | 360 (313, 404.75) | 0.012 |

Additional file 2: Table S3. The expression of DEPs in IgAN.

| Protein Accession | Protein | Protein Names | log2FC | adj.pvalue | Discription |
| --- | --- | --- | --- | --- | --- |
| Q8IUW5 | RELL1 | RELT-like protein 1 | 2.984 | 0.033 | UP |
| Q96HN2 | AHCYL2 | Adenosylhomocysteinase 3 | 2.065 | 0.040 | UP |
| Q9NUN5 | LMBRD1 | Lysosomal cobalamin transport escort protein LMBD1 | 1.628 | 0.001 | UP |
| Q2TAA2 | IAH1 | Isoamyl acetate-hydrolyzing esterase 1 homolog | 1.118 | 0.018 | UP |
| Q9P2N5 | RBM27 | RNA-binding protein 27 | 1.087 | 0.000 | UP |
| Q9UKU7 | ACAD8 | Acyl-CoA dehydrogenase family member 8 | 0.958 | 0.003 | UP |
| P0DP01 | IGHV1-8 | Immunoglobulin heavy variable 1-8 | 0.948 | 0.029 | UP |
| O75911 | DHRS3 | Short-chain dehydrogenase/reductase 3 | 0.946 | 0.006 | UP |
| Q00889 | PSG6 | Pregnancy-specific beta-1-glycoprotein 6 | 0.934 | 0.048 | UP |
| Q9P2E2 | KIF17 | Kinesin-like protein KIF17 | 0.893 | 0.036 | UP |
| Q7L5Y1 | ENOSF1 | Mitochondrial enolase superfamily member 1 | 0.885 | 0.001 | UP |
| A5D8V6 | VPS37C | Vacuolar protein sorting-associated protein 37C | 0.847 | 0.000 | UP |
| Q8NHP8 | PLBD2 | Putative phospholipase B-like 2 | 0.818 | 0.032 | UP |
| Q9Y5J7 | TIMM9 | Mitochondrial import inner membrane translocase subunit Tim9 | 0.812 | 0.000 | UP |
| Q6UWY5 | OLFML1 | Olfactomedin-like protein 1 | 0.796 | 0.000 | UP |
| Q5VW36 | FOCAD | Focadhesin | 0.792 | 0.043 | UP |
| O43166 | SIPA1L1 | Signal-induced proliferation-associated 1-like protein 1 | 0.789 | 0.017 | UP |
| Q13825 | AUH | AU-binding protein/enoyl-CoA hydratase | 0.759 | 0.006 | UP |
| P40189 | IL6ST | Interleukin-6 receptor subunit beta | 0.717 | 0.000 | UP |
| Q6PKG0 | LARP1 | La-related protein 1 | 0.702 | 0.043 | UP |
| P49815 | TSC2 | Tuberous sclerosis 2 protein | 0.693 | 0.045 | UP |
| P61769 | B2M | Beta-2-microglobulin | 0.668 | 0.042 | UP |
| Q9BV10 | ALG12 | Dol-P-Man:Man(7)GlcNAc(2)-PP-Dol alpha-1,6-mannosyltransferase | 0.603 | 0.043 | UP |
| Q15036 | SNX17 | Sorting nexin-17 | 0.586 | 0.001 | UP |
| P34913 | EPHX2 | Bifunctional epoxide hydrolase 2 | 0.496 | 0.010 | UP |
| P36980 | CFHR2 | Complement factor H-related protein 2 | 0.492 | 0.045 | UP |
| Q8TD55 | PLEKHO2 | Pleckstrin homology domain-containing family O member 2 | 0.474 | 0.003 | UP |
| P15309 | ACP3 | Protein tyrosine phosphatase ACP3 | 0.463 | 0.033 | UP |
| P02750 | LRG1 | Leucine-rich alpha-2-glycoprotein | 0.447 | 0.004 | UP |
| P36897 | TGFBR1 | TGF-beta receptor type-1 | 0.437 | 0.049 | UP |
| P02743 | APCS | Serum amyloid P-component | 0.387 | 0.007 | UP |
| P32119 | PRDX2 | Peroxiredoxin-2 | 0.365 | 0.010 | UP |
| P05187 | ALPP | Alkaline phosphatase, placental type | 0.334 | 0.018 | UP |
| P02748 | C9 | Complement component C9 | 0.309 | 0.029 | UP |
| P02749 | APOH | Apolipoprotein H | 0.280 | 0.008 | UP |
| Q9Y2L1 | DIS3 | Protein DIS3 homolog | 0.255 | 0.045 | UP |
| P20851 | C4BPB | C4b-binding protein beta chain | 0.249 | 0.008 | UP |
| P07225 | PROS1 | Vitamin K-dependent protein S | 0.194 | 0.043 | UP |
| P08603 | CFH | Complement factor H | 0.190 | 0.040 | UP |
| P01031 | C5 | Complement C5 | 0.171 | 0.043 | UP |
| P02768 | ALB | Albumin | -0.173 | 0.008 | Down |
| P00450 | CP | Ceruloplasmin | -0.173 | 0.029 | Down |
| Q16610 | ECM1 | Extracellular matrix protein 1 | -0.203 | 0.037 | Down |
| P51451 | BLK | Tyrosine-protein kinase Blk | -0.256 | 0.001 | Down |
| P06870 | KLK1 | Kallikrein-1 | -0.302 | 0.010 | Down |
| Q9NZJ4 | SACS | Sacsin | -0.309 | 0.007 | Down |
| Q9NP73 | ALG13 | Asparagine-linked glycosylation 13 homolog | -0.311 | 0.000 | Down |
| Q16740 | CLPP | ATP-dependent Clp protease proteolytic subunit, mitochondrial | -0.319 | 0.034 | Down |
| P47989 | XDH | Xanthine dehydrogenase | -0.321 | 0.000 | Down |
| P23083 | IGHV1-2 | Immunoglobulin heavy variable 1-2 | -0.329 | 0.033 | Down |
| Q07889 | SOS1 | Son of sevenless homolog 1 | -0.335 | 0.000 | Down |
| Q8NFI4 | ST13P5 | Suppression of tumorigenicity 13 pseudogene 5 | -0.341 | 0.042 | Down |
| Q969G3 | SMARCE1 | SWI/SNF-related matrix-associated actin-dependent regulator of chromatin subfamily E member 1 | -0.357 | 0.015 | Down |
| P13861 | PRKAR2A | cAMP-dependent protein kinase type II-alpha regulatory subunit | -0.368 | 0.005 | Down |
| A0A0C4DH31 | IGHV1-18 | Immunoglobulin heavy variable 1-18 | -0.383 | 0.000 | Down |
| P11177 | PDHB | Pyruvate dehydrogenase E1 component subunit beta, mitochondrial | -0.386 | 0.029 | Down |
| Q9Y624 | F11R | Junctional adhesion molecule A | -0.390 | 0.043 | Down |
| Q15691 | MAPRE1 | Microtubule-associated protein RP/EB family member 1 | -0.424 | 0.043 | Down |
| P68871 | HBB | Hemoglobin subunit beta | -0.458 | 0.032 | Down |
| P69905 | HBA1 | Hemoglobin subunit alpha | -0.490 | 0.010 | Down |
| O60884 | DNAJA2 | DnaJ homolog subfamily A member 2 | -0.516 | 0.028 | Down |
| Q9H668 | STN1 | CST complex subunit STN1 | -0.522 | 0.012 | Down |
| Q13614 | MTMR2 | Myotubularin-related protein 2 | -0.539 | 0.011 | Down |
| Q9UBW8 | COPS7A | COP9 signalosome complex subunit 7a | -0.558 | 0.029 | Down |
| Q96E39 | RBMXL1 | RNA binding motif protein, X-linked-like-1 | -0.630 | 0.036 | Down |
| P29972 | AQP1 | Aquaporin-1 | -0.719 | 0.015 | Down |
| P40692 | MLH1 | MutL protein homolog 1 | -0.893 | 0.028 | Down |
| Q14534 | SQLE | Squalene monooxygenase | -1.052 | 0.041 | Down |
| O14562 | UBFD1 | Ubiquitin domain-containing protein UBFD1 | -1.331 | 0.007 | Down |
| Q9UP38 | FZD1 | Frizzled-1 | -1.529 | 0.036 | Down |
| Q15061 | WDR43 | WD repeat-containing protein 43 | -1.731 | 0.008 | Down |
|  |  |  |  |  |  |

Additional file 2: Table S4. The top 10 GO BP analysis of upregulate and downregulate in DEPs of IgAN.

| GO_BP_top10_up_result | | | | | |
| --- | --- | --- | --- | --- | --- |
| ID | Description | pvalue | GeneID | Count | -LOG10(pvalue) |
| GO:0006956 | complement activation | 2.060E-09 | IGHV1-8/CFHR2/APCS/  C9/C4BPB/PROS1/CFH/C5 | 8 | 8.685E+00 |
| GO:0030449 | regulation of complement activation | 1.060E-07 | CFHR2/C9/  C4BPB/PROS1/CFH/C5 | 6 | 6.975E+00 |
| GO:0002920 | regulation of humoral immune response | 3.130E-07 | CFHR2/C9/  C4BPB/PROS1/CFH/C5 | 6 | 6.504E+00 |
| GO:0006959 | humoral immune response | 7.270E-07 | IGHV1-8/CFHR2/  APCS/C9/C4BPB/  PROS1/CFH/C5 | 8 | 6.139E+00 |
| GO:0006957 | complement activation, alternative pathway | 6.030E-06 | C9/CFH/C5 | 3 | 5.220E+00 |
| GO:0006958 | complement activation, classical pathway | 8.950E-06 | IGHV1-8/APCS/  C9/C4BPB/C5 | 5 | 5.048E+00 |
| GO:0002455 | humoral immune response mediated by circulating immunoglobulin | 1.460E-05 | IGHV1-8/APCS/  C9/C4BPB/C5 | 5 | 4.835E+00 |
| GO:0019835 | cytolysis | 3.260E-05 | CFHR2/C9/C5 | 3 | 4.487E+00 |
| GO:0002697 | regulation of immune effector process | 3.440E-05 | B2M/CFHR2/C9/  C4BPB/PROS1/CFH/C5 | 7 | 4.463E+00 |
| GO:0002449 | lymphocyte mediated immunity | 7.640E-05 | IGHV1-8/B2M/  APCS/C9/C4BPB/C5 | 6 | 4.117E+00 |
|  |  |  |  |  |  |

| GO_BP_top10_down_result | | | | | |
| --- | --- | --- | --- | --- | --- |
| ID | Description | pvalue | geneID | Count | -LOG10(pvalue) |
| GO:0015669 | gas transport | 3.660E-06 | HBB/HBA1/AQP1 | 3 | 5.437E+00 |
| GO:0015701 | bicarbonate transport | 3.870E-05 | HBB/HBA1/AQP1 | 3 | 4.412E+00 |
| GO:0070527 | platelet aggregation | 1.163E-04 | BLK/F11R/HBB | 3 | 3.935E+00 |
| GO:0015671 | oxygen transport | 2.367E-04 | HBB/HBA1 | 2 | 3.626E+00 |
| GO:0034109 | homotypic cell-cell adhesion | 2.965E-04 | BLK/F11R/HBB | 3 | 3.528E+00 |
| GO:0090330 | regulation of platelet aggregation | 3.840E-04 | BLK/F11R | 2 | 3.416E+00 |
| GO:0098869 | cellular oxidant detoxification | 5.065E-04 | ALB/HBB/HBA1 | 3 | 3.295E+00 |
| GO:1990748 | cellular detoxification | 7.186E-04 | ALB/HBB/HBA1 | 3 | 3.143E+00 |
| GO:0003014 | renal system process | 7.745E-04 | PRKAR2A/HBB/AQP1 | 3 | 3.111E+00 |
| GO:0034110 | regulation of homotypic cell-cell adhesion | 7.823E-04 | BLK/F11R | 2 | 3.107E+00 |
|  |  |  |  |  |  |

Additional file 2: Table S5. The expression of DAMs in IgAN.

| Compound | Super Class | HMDB | VIP | Fold Change | log2(FC) | p.value |
| --- | --- | --- | --- | --- | --- | --- |
| (3E,5Z)-1,3,5-Heptatriene | Hydrocarbons | HMDB0061889 | 1.428 | 0.603 | -0.729 | 2.495E-06 |
| (3Z,6E)-N-Hydroxy-2,4,4,7-tetramethyl-6,8-nonadien-3-imine | Organic nitrogen compounds | HMDB0036031 | 1.431 | 1.544 | 0.627 | 1.194E-06 |
| (E,E)-2,4-Dodecadienal | Organic oxygen compounds | HMDB0039590 | 1.507 | 0.485 | -1.044 | 2.472E-06 |
| (E,E)-2,4-Heptadienal | Organic oxygen compounds | HMDB0303844 | 1.100 | 1.343 | 0.426 | 6.371E-04 |
| (R)-3-Hydroxy-tetradecanoic acid | Lipids and lipid-like molecules | HMDB0010731 | 1.107 | 0.590 | -0.762 | 2.655E-05 |
| (S)-(+)-2-Aminobutyric Acid | Organic acids and derivatives | HMDB0000452 | 1.229 | 1.567 | 0.648 | 2.190E-03 |
| (S)-2-methylbutanal | Organic oxygen compounds | HMDB0031525 | 1.604 | 0.354 | -1.498 | 2.213E-08 |
| 10-Hydroxy-2-decenoic acid | Organic acids and derivatives | HMDB0244269 | 1.484 | 0.487 | -1.039 | 1.170E-05 |
| 12-Hydroxystearic acid | Lipids and lipid-like molecules | HMDB0061706 | 1.367 | 0.330 | -1.599 | 1.714E-05 |
| 12-Hydroxylauric acid | Organic acids and derivatives | HMDB0002059 | 1.360 | 0.436 | -1.197 | 3.498E-05 |
| 2-(14,15-Epoxyeicosatrienoyl) Glycerol | Lipids and lipid-like molecules | HMDB0013651 | 1.325 | 0.652 | -0.616 | 7.378E-05 |
| 2,4,7-Decatrienoic acid | Lipids and lipid-like molecules | HMDB0035235 | 1.534 | 0.303 | -1.725 | 1.965E-08 |
| 2-[4-(3-Hydroxypropyl)-2-methoxyphenoxy]-1,3-propanediol | Benzenoids | HMDB0040352 | 1.558 | 0.312 | -1.683 | 7.879E-08 |
| 2-Ethyltoluene | Benzenoids | HMDB0059819 | 1.082 | 1.365 | 0.448 | 7.251E-04 |
| 2-Hydroxycinnamic acid | Phenylpropanoids and polyketides | HMDB0002641 | 1.604 | 0.386 | -1.373 | 5.433E-08 |
| 2-Hydroxymyristic acid | Lipids and lipid-like molecules | HMDB0002261 | 1.341 | 0.406 | -1.300 | 1.650E-05 |
| 2-Mercaptoethanol | Organosulfur compounds | HMDB0245190 | 1.096 | 1.498 | 0.583 | 1.144E-03 |
| 2-Methyl-3-thioacetoxy-4,5-dihydrofuran | Organoheterocyclic compounds | HMDB0037786 | 1.820 | 0.301 | -1.733 | 1.276E-10 |
| 2-Nonenoic acid | Lipids and lipid-like molecules | HMDB0031271 | 1.259 | 0.431 | -1.213 | 1.346E-05 |
| 3,5-di-tert-Butyl-4-hydroxybenzaldehyde | Organic oxygen compounds | HMDB0240643 | 1.254 | 1.405 | 0.491 | 3.824E-04 |
| 3-Hydroxybutyric acid | Organic acids and derivatives | HMDB0000357 | 1.307 | 1.956 | 0.968 | 5.413E-05 |
| 3-Methylcyclopentene | Hydrocarbons | HMDB0031544 | 1.363 | 1.444 | 0.530 | 1.145E-05 |
| 5-(Heptadec-12-enyl)resorcinol | Benzenoids | HMDB0038527 | 1.567 | 0.520 | -0.943 | 1.112E-05 |
| 5-Hydroxyindoleacetic acid | Organoheterocyclic compounds | HMDB0000763 | 1.437 | 1.725 | 0.787 | 1.660E-05 |
| 5-Nonyltetrahydro-2-oxo-3-furancarboxylic acid | Organoheterocyclic compounds | HMDB0030993 | 1.161 | 0.554 | -0.851 | 2.124E-04 |
| 5-Phenylvaleric acid | Lipids and lipid-like molecules | HMDB0002043 | 1.380 | 0.565 | -0.824 | 1.970E-06 |
| 6-keto-Prostaglandin F1a | Lipids and lipid-like molecules | HMDB0002886 | 1.508 | 0.313 | -1.674 | 3.437E-07 |
| 9-Oxononanoic acid | Lipids and lipid-like molecules | HMDB0094711 | 1.136 | 0.653 | -0.615 | 9.729E-04 |
| Ac-Ala-OH | Organic acids and derivatives | HMDB0000766 | 1.196 | 1.435 | 0.521 | 3.064E-04 |
| Acrimarine H | Organoheterocyclic compounds | HMDB0038599 | 1.090 | 0.472 | -1.084 | 5.802E-04 |
| Acrylic acid | Organic acids and derivatives | HMDB0031647 | 1.203 | 1.639 | 0.713 | 1.743E-04 |
| asp-leu | Organic acids and derivatives | HMDB0028757 | 1.472 | 1.768 | 0.822 | 1.818E-05 |
| Aspulvinone H | Benzenoids | HMDB0034472 | 1.407 | 2.660 | 1.412 | 1.062E-04 |
| Buclizine | Benzenoids | HMDB0014498 | 1.087 | 1.356 | 0.439 | 1.071E-03 |
| Cinnamic acid | Phenylpropanoids and polyketides | HMDB0000567 | 1.321 | 1.396 | 0.481 | 3.426E-05 |
| Creatine | Organic acids and derivatives | HMDB0000064 | 1.722 | 2.031 | 1.022 | 5.883E-09 |
| Creatinine | Organic acids and derivatives | HMDB0000562 | 1.609 | 2.014 | 1.010 | 4.158E-07 |
| D-(+)-Tryptophan | Organoheterocyclic compounds | HMDB0013609 | 1.290 | 1.444 | 0.530 | 9.846E-05 |
| Dihydrothymine | Organoheterocyclic compounds | HMDB0000079 | 1.207 | 2.772 | 1.471 | 4.951E-04 |
| DL-Carnitine | Organic nitrogen compounds | HMDB0000062 | 1.352 | 1.552 | 0.634 | 4.738E-05 |
| Docosapentaenoic acid | Lipids and lipid-like molecules | HMDB0006528 | 1.471 | 0.518 | -0.948 | 5.178E-05 |
| Dodecanedioic acid | Lipids and lipid-like molecules | HMDB0000623 | 1.023 | 0.611 | -0.712 | 3.841E-03 |
| Dodecenoic acid | Lipids and lipid-like molecules | HMDB0032248 | 1.555 | 0.436 | -1.197 | 1.077E-06 |
| D-PANTOTHENIC ACID | Organic oxygen compounds | HMDB0000210 | 1.034 | 1.112 | 0.153 | 9.114E-03 |
| Eicosanedioic acid | Lipids and lipid-like molecules | HMDB0242141 | 1.030 | 0.619 | -0.692 | 2.453E-03 |
| Ethyl acetate | Organic acids and derivatives | HMDB0031217 | 1.325 | 1.589 | 0.668 | 1.218E-04 |
| Ethyl furfurylacetate | Lipids and lipid-like molecules | HMDB0032921 | 1.273 | 0.542 | -0.883 | 2.222E-05 |
| Gabapentin | Organic acids and derivatives | HMDB0005015 | 1.058 | 1.781 | 0.832 | 4.458E-03 |
| Glycerol tripropanoate | Lipids and lipid-like molecules | HMDB0032857 | 1.308 | 0.682 | -0.552 | 3.931E-05 |
| Hexadecanamide | Lipids and lipid-like molecules | HMDB0012273 | 1.468 | 1.447 | 0.533 | 5.965E-07 |
| Hexylbenzene | Benzenoids | HMDB0061815 | 1.010 | 1.312 | 0.392 | 3.037E-03 |
| Hydroxyhexanoycarnitine | Lipids and lipid-like molecules | HMDB0013131 | 1.171 | 0.630 | -0.667 | 3.916E-05 |
| Indoleacrylic acid | Organoheterocyclic compounds | HMDB0000734 | 1.293 | 1.444 | 0.530 | 9.551E-05 |
| Kynurenine | Organic oxygen compounds | HMDB0000684 | 1.216 | 2.579 | 1.367 | 5.141E-05 |
| L-(-)-Methionine | Organic acids and derivatives | HMDB0000696 | 1.239 | 1.703 | 0.768 | 1.742E-04 |
| L-(+)-Lactic acid | Organic acids and derivatives | HMDB0000190 | 1.072 | 1.768 | 0.822 | 7.852E-04 |
| Lauramide | Lipids and lipid-like molecules | HMDB0251566 | 1.588 | 1.517 | 0.601 | 1.526E-07 |
| L-gamma-Glutamyl-L-leucine | Organic acids and derivatives | HMDB0011171 | 1.078 | 1.233 | 0.302 | 5.766E-03 |
| L-Histidine | Organic acids and derivatives | HMDB0000177 | 1.439 | 1.721 | 0.783 | 7.293E-07 |
| Linoleamide | Lipids and lipid-like molecules | HMDB0062656 | 1.466 | 1.455 | 0.541 | 2.375E-06 |
| Linoleyl carnitine | Lipids and lipid-like molecules | HMDB0006469 | 1.060 | 1.524 | 0.608 | 1.142E-03 |
| L-Norleucine | Organic acids and derivatives | HMDB0001645 | 1.495 | 1.628 | 0.703 | 3.815E-06 |
| L-Phenylalanine | Organic acids and derivatives | HMDB0000159 | 1.605 | 1.719 | 0.782 | 1.474E-07 |
| LysoPE(16:0/0:0) | Lipids and lipid-like molecules | HMDB0011503 | 1.350 | 1.545 | 0.628 | 4.833E-05 |
| Methyl acetoacetate | Organic acids and derivatives | HMDB0000310 | 1.130 | 1.570 | 0.650 | 1.833E-04 |
| Mono(2-ethylhexyl) phthalate (MEHP) | Benzenoids | HMDB0013248 | 1.455 | 1.949 | 0.962 | 2.775E-07 |
| Myricanone | Phenylpropanoids and polyketides | HMDB0030798 | 1.582 | 2.234 | 1.160 | 3.831E-06 |
| N-(3-acetamidopropyl)pyrrolidin-2-one | Organoheterocyclic compounds | HMDB0061384 | 1.419 | 1.720 | 0.782 | 1.928E-05 |
| N-Acetylisoputreanine | Organic acids and derivatives | HMDB0094713 | 1.223 | 1.704 | 0.769 | 7.935E-04 |
| Naratriptan | Organoheterocyclic compounds | HMDB0015087 | 1.238 | 0.496 | -1.012 | 1.013E-04 |
| Ornithine | Organic acids and derivatives | HMDB0000214 | 1.050 | 1.466 | 0.552 | 2.944E-03 |
| Palmitoleic acid | Lipids and lipid-like molecules | HMDB0003229 | 1.688 | 0.341 | -1.552 | 5.711E-09 |
| Paraldehyde | Organoheterocyclic compounds | HMDB0032456 | 1.588 | 1.693 | 0.760 | 8.292E-08 |
| Phosphoric acid | Homogeneous non-metal compounds | HMDB0002142 | 1.323 | 3.117 | 1.640 | 8.459E-06 |
| Polyethylene, oxidized; | Organic acids and derivatives | HMDB0032472 | 1.536 | 0.408 | -1.295 | 1.916E-07 |
| Postin | Organic acids and derivatives | HMDB0005772 | 1.530 | 2.063 | 1.045 | 6.542E-07 |
| Pravastatin | Organic acids and derivatives | HMDB0005022 | 1.211 | 0.385 | -1.377 | 1.020E-04 |
| Pro-leu | Organic acids and derivatives | HMDB0253028 | 1.365 | 1.865 | 0.899 | 7.833E-05 |
| Prolinamide | Organic acids and derivatives | HMDB0253910 | 1.457 | 1.607 | 0.685 | 1.579E-05 |
| prostaglandin G2 2-glyceryl ester | Lipids and lipid-like molecules | HMDB0062591 | 1.126 | 0.499 | -1.002 | 4.708E-04 |
| Sebacic acid | Lipids and lipid-like molecules | HMDB0000792 | 1.107 | 0.611 | -0.712 | 5.112E-04 |
| Suberic acid | Lipids and lipid-like molecules | HMDB0000893 | 1.477 | 0.369 | -1.439 | 5.471E-07 |
| Succinic semialdehyde | Lipids and lipid-like molecules | HMDB0001259 | 1.078 | 1.378 | 0.462 | 1.600E-03 |
| trans-3-Hexenoic acid | Lipids and lipid-like molecules | HMDB0031501 | 1.494 | 1.631 | 0.706 | 3.864E-06 |
| Traumatic Acid | Lipids and lipid-like molecules | HMDB0000933 | 1.145 | 1.739 | 0.798 | 7.922E-04 |
| Tricaprilin | Lipids and lipid-like molecules | HMDB0011187 | 1.489 | 0.441 | -1.182 | 3.453E-05 |
| Valdecoxib | Benzenoids | HMDB0005033 | 1.099 | 2.444 | 1.289 | 6.396E-04 |
| Vanillyl alcohol | Benzenoids | HMDB0032012 | 1.424 | 0.517 | -0.951 | 4.878E-07 |
| Varanic acid | Lipids and lipid-like molecules | HMDB0002195 | 1.584 | 0.427 | -1.226 | 5.902E-06 |
| xi-8-Hydroxyhexadecanedioic acid | Lipids and lipid-like molecules | HMDB0037831 | 1.255 | 2.070 | 1.050 | 5.711E-05 |
|  |  |  |  |  |  |  |

Additional file 2: Table S6. The pathway of DAMs enrichment in IgAN.

| Pathway | compound | p-value | FDR |
| --- | --- | --- | --- |
| Glycine, serine and threonine metabolism | Creatine | 4.170E-09 | 7.100E-08 |
| Tryptophan metabolism | 5-Hydroxyindoleacetate; | 2.030E-08 | 1.730E-07 |
| Arginine and proline metabolism | Creatine | 5.650E-08 | 2.470E-07 |
| Aminoacyl-tRNA biosynthesis | L-Histidine | 5.820E-08 | 2.470E-07 |
| Phenylalanine, tyrosine and tryptophan biosynthesis | L-Phenylalanine | 2.480E-07 | 7.030E-07 |
| Phenylalanine metabolism | L-Phenylalanine | 2.480E-07 | 7.030E-07 |
| Histidine metabolism | L-Histidine | 3.500E-06 | 7.430E-06 |
| beta-Alanine metabolism | L-Histidine | 3.500E-06 | 7.430E-06 |
| Butanoate metabolism | (R)-3-Hydroxybutanoate | 1.470E-05 | 2.770E-05 |
| Synthesis and degradation of ketone bodies | (R)-3-Hydroxybutanoate | 4.130E-05 | 7.030E-05 |
| Cysteine and methionine metabolism | L-Methionine | 1.375E-04 | 2.125E-04 |
| Glycolysis / Gluconeogenesis | (S)-Lactate; | 7.602E-04 | 9.941E-04 |
| Pyruvate metabolism | (S)-Lactate | 7.602E-04 | 9.941E-04 |
| Alanine, aspartate and glutamate metabolism | Succinate semialdehyde | 1.446E-03 | 1.756E-03 |
| Arginine biosynthesis | L-Ornithine | 2.093E-03 | 2.224E-03 |
| Glutathione metabolism | L-Ornithine | 2.093E-03 | 2.224E-03 |
| Pantothenate and CoA biosynthesis | Pantothenate | 5.885E-03 | 5.885E-03 |

Additional file 2: TableS7. Clinical characteristics of the patients with IgAN and normal control in the validation cohort.

| Characteristic | Health | IgAN | p |
| --- | --- | --- | --- |
| n | 10 | 9 |  |
| gender, n (%) |  |  | 1.000 |
| F | 8 (42.1%) | 7 (36.8%) |  |
| M | 2 (10.5%) | 2 (10.5%) |  |
| age, median (IQR) | 35 (33.25, 39) | 50 (43, 59) | 0.270 |
| CRE, median (IQR) | 60 (58, 65) | 71.8 (68.9, 194.8) | 0.060 |
| URE, median (IQR) | 3.36 (3.08, 3.38) | 5.95 (5.22, 8.27) | < 0.001 |
| UA, mean ± SD | 222.2 ± 50.41 | 347.22 ± 101.66 | 0.026 |
